# RNA-Seq transcriptome analysis of *Amaranthus palmeri* with differential tolerance to glufosinate herbicide

**DOI:** 10.1371/journal.pone.0195488

**Published:** 2018-04-19

**Authors:** Reiofeli A. Salas-Perez, Christopher A. Saski, Rooksana E. Noorai, Subodh K. Srivastava, Amy L. Lawton-Rauh, Robert L. Nichols, Nilda Roma-Burgos

**Affiliations:** 1 Department of Crop, Soil, and Environmental Sciences, University of Arkansas, Fayetteville, Arkansas, United States of America; 2 Department of Genetics and Biochemistry, Clemson University, Clemson, South Carolina, United States of America; 3 Cotton Incorporated, Cary, North Carolina, United States of America; National Taiwan University, TAIWAN

## Abstract

*Amaranthus palmeri* (Amaranthaceae) is a noxious weed in several agroecosystems and in some cases seriously threatens the sustainability of crop production in North America. Glyphosate-resistant *Amaranthus* species are widespread, prompting the use of alternatives to glyphosate such as glufosinate, in conjunction with glufosinate-resistant crop cultivars, to help control glyphosate-resistant weeds. An experiment was conducted to analyze the transcriptome of *A*. *palmeri* plants that survived exposure to 0.55 kg ha^-1^ glufosinate. Since there was no record of glufosinate use at the collection site, survival of plants within the population are likely due to genetic expression that pre-dates selection; in the formal parlance of weed science this is described as natural tolerance. Leaf tissues from glufosinate-treated and non-treated seedlings were harvested 24 h after treatment (HAT) for RNA-Seq analysis. Global gene expression was measured using Illumina DNA sequence reads from non-treated and treated surviving (presumably tolerant, T) and susceptible (S) plants. The same plants were used to determine the mechanisms conferring differential tolerance to glufosinate. The S plants accumulated twice as much ammonia as did the T plants, 24 HAT. The relative copy number of the glufosinate target gene *GS2* did not differ between T and S plants, with 1 to 3 *GS2* copies in both biotypes. A reference cDNA transcriptome consisting of 72,780 contigs was assembled, with 65,282 sequences putatively annotated. Sequences of *GS2* from the transcriptome assembly did not have polymorphisms unique to the tolerant plants. Five hundred sixty-seven genes were differentially expressed between treated T and S plants. Of the upregulated genes in treated T plants, 210 were more highly induced than were the upregulated genes in the treated S plants. Glufosinate-tolerant plants had greater induction of ABC transporter, glutathione S-transferase (*GST*), NAC transcription factor, nitronate monooxygenase (*NMO*), chitin elicitor receptor kinase (*CERK1*), heat shock protein 83, ethylene transcription factor, heat stress transcription factor, NADH-ubiquinone oxidoreductase, ABA 8’-hydroxylase, and cytochrome P450 genes (*CYP72A*, *CYP94A1*). Seven candidate genes were selected for validation using quantitative real time-PCR. While *GST* was upregulated in treated tolerant plants in at least one population, *CYP72A219* was consistently highly expressed in all treated tolerant biotypes. These genes are candidates for contributing tolerance to glufosinate. Taken together, these results show that differential induction of stress-protection genes in a population can enable some individuals to survive herbicide application. Elevated expression of detoxification-related genes can get fixed in a population with sustained selection pressure, leading to evolution of resistance. Alternatively, sustained selection pressure could select for mutation(s) in the *GS2* gene with the same consequence.

## Introduction

*Amaranthus palmeri* is a dioecious, weedy *Amaranthus* species native to Southwestern North America [1, 2]. It is one of the most widespread, troublesome, and economically damaging weeds in agronomic crops throughout the southern United States [2]. Infestation of Palmer amaranth can cause from 70% to more than 90% yield loss in cotton (*Gossypium hirsutum*) [3], soybean (*Glycine max*) [4], and corn (*Zea mays*) [5]. *A*. *palmeri* is difficult to control because of its rapid growth rate, high fecundity, tiny seeds dispersed by multiple agents, continuous emergence pattern, high genetic diversity, high propensity for evolving herbicide resistance, and dioecious nature with long-distance pollen dispersal [1, 6–9]. To date, resistances to six herbicide mechanisms of action (MOAs) have been confirmed in *A*. *palmeri*: acetolactate synthase (ALS) inhibitors, carotenoid biosynthesis (4-hydroxyphenylpyruvate dioxygenase (HPPD) inhibitors, enolpyruvyl shikimate-3-phosphate synthase (EPSPS) inhibitor (glyphosate), mitosis inhibitors (dinitroanilines), photosystem II inhibitors (triazines), and protoporphyrinogen oxidase (PPO) inhibitors [10]. The increasing resistance of *A*. *palmeri* to herbicides is a threat to corn, cotton, peanut (*Arachis hypogea*), and soybean production [1, 11–14]. Alternative management strategies are needed to combat this problem in several areas in North America [11, 15–18].

Herbicides are used as a major tool for controlling weeds and the evolution of herbicide-resistant (HR) weeds is an increasing issue worldwide. Glyphosate-resistant (GR) crops, first commercialized in 1996, were adopted quickly by growers because the technology allowed the use of the nonselective herbicide, glyphosate, in-season. The technology drastically simplified weed control with the use of a single, inexpensive, highly effective herbicide. In fact, GR crops constituted 80% of the 175 million ha planted with transgenic crops globally [19]. However, the over-reliance on glyphosate and its application over a vast land area has exerted unprecedented selection pressure on weeds, resulting in the evolution of GR weeds including *A*. *palmeri*. Glyphosate-resistant *A*. *palmeri* was first identified in Georgia in 2004 [20] and subsequently became widespread across the South, Midwest and Northeast regions of the United States [10]. The widespread distribution of glyphosate-resistant weeds compelled farmers to use alternative herbicides including another non-selective herbicide, glufosinate, to control HR weeds in glufosinate-tolerant crops. Glufosinate is a fast-acting postemergence herbicide that controls weeds by inhibiting glutamine synthetase (GS) (E.C. 6.3.1.2), the enzyme that converts glutamate and ammonia to glutamine [21]. Inhibition of GS by glufosinate leads to ammonia accumulation, inhibition of amino acid synthesis, and indirect inhibition of photosynthesis, ultimately leading to plant death [22]. To date, resistance to glufosinate has been confirmed in *Eleusine indica* from Malaysia [23, 24] and *Lolium perenne* ssp. *multiflorum* from Oregon [25]. An amino acid mutation in the chloroplast-encoded GS gene, Asp_171_Asn, conferred resistance to glufosinate in *L*. *perenne* ssp. *multiflorum* [26]. Resistance to glufosinate in glufosinate-resistant crops is achieved using transgenic methods to insert the *bar* or *pat* (phosphinothricin acetyltransferase) gene from a bacterium to the plant’s genome, allowing detoxification of glufosinate by acetylation [27].

Differential responses to glufosinate in weeds have been attributed to several factors including light, temperature, humidity, growth stage, application rate, application timing, species, and variation in the level of herbicide absorption, translocation, and metabolism [28, 29]. Control of *A*. *palmeri* by glufosinate is variable [30–32]. A previous study reported higher uptake, mobility, and metabolism of glufosinate in *A*. *palmeri* compared to the more susceptible *Ipomoea lacunosa* [28]. As is commonly observed, differential tolerance to herbicides are often due to non-target-site (NTS) mechanisms, involving the detoxification of herbicide by biochemical modification and/or the compartmentation of the herbicide and its metabolites [33]. Cases of weed resistance to herbicides due to NTS mechanisms have been increasing (www.weedscience.org). The genetic bases of NTS mechanisms are not fully understood due to the complex interaction of biochemical processes and limited genomic information on weedy species [33–35]. In this study, we investigated *A*. *palmeri* accessions with differential tolerance to glufosinate.

The genome of *A*. *palmeri* is not yet deciphered although the genome and transcriptome of its cultivated relative grain amaranth (*Amaranthus hypochondriacus*) was completed in 2014 [36]. Also recently, the transcriptome of two weedy species *Lolium rigidum* [37] and *Echinochloa crus-galli* [38] were sequenced to identify genes involved in herbicide resistance. Understanding the molecular mechanisms underlying herbicide resistance could be used to mitigate and manage resistance evolution and reduce weed impact on crops. This study assembled the transcriptome sequence of *A*. *palmeri* exposed to glufosinate compared to controls to elucidate candidate genes involved in differential tolerance to glufosinate.

## Materials and methods

### Plant materials

*Amaranthus palmeri* seed samples from 120 fields were collected in Arkansas, USA between 2008 and 2014. The collection of plant samples from the field was done with permission of farm owners, managers, consultants, or Extension Agents. In the process of collecting samples, no endangered species were affected. Inflorescences of at least 10 female plants per field were harvested, dried, threshed, and cleaned for herbicide bioassays in the greenhouse. One accession of interest (08-Lee-C) was collected from a field that was planted with glyphosate-tolerant (Roundup Ready^®^) soybean in 2008 and glyphosate-tolerant cotton in 2006 and 2007. Although this field had no record of being sprayed with glufosinate, some plants survived exposure to glufosinate (0.55 kg ha^-1^) in the greenhouse. The survivors were grown and allowed to cross-pollinate to produce the first cycle of purified (intercrossed) progeny (C1).

To study the potential survival mechanisms, seeds of 08-Lee-C and the C1 progeny were planted in 4-cm-diameter pots using commercial potting soil mix (Sunshine Mix, Bellevue, WA, USA). Seedlings (100) were grown at one plant per pot in a growth chamber maintained at 32/26 °C day/night temperature with a 16-h photoperiod. Plants were watered daily and fertilized with a water-soluble all-purpose plant food containing 15-30-15% NPK (Miracle-Gro^®^, Marysville, OH, USA), every 2 wk. Fifty plants per accession (9-cm tall) were sprayed with glufosinate (0.55 kg ai ha^-1^) (Liberty^®^, Bayer) mixed with 20 g L^-1^ ammonium sulfate to identify S and T plants. Susceptible and T plants from the non-treated control were identified by ammonia accumulation assay. Six confirmed S plants from 08-Lee-C and T plants from C1 were used for ammonia accumulation assay, determination of chloroplast-encoded glutamine synthetase (*GS2*) copy number, and RNA-Seq experiment.

### Phenotypic evaluation of *A*. *palmeri* response to glufosinate

The response of *A*. *palmeri*, collected between 2008 and 2014, was evaluated in the greenhouse. A known herbicide-susceptible accession (SS) was included in each experiment as control [39]. Five-hundred mg of seeds from each field-collected plant were mixed to make a composite seed sample representing each accession. The experiment was conducted twice in a randomized complete block design with two replications. Each replication consisted of one cellular tray (28 X 54 cm) with 50 cells (Redway Feed Garden and Pet Supply, Reedway, CA, USA) filled with a commercial medium (Sunshine Mix, Bellevue, WA). Composite seeds from each accession were planted in each cell and seedlings were thinned to one per cell. Glufosinate was applied at 0.55 kg ha^-1^ when seedlings were 7.5–9 cm tall using a laboratory sprayer fitted with a flat fan nozzle tip (800067 TeeJet, Spraying Systems Co., Wheaton, IL, USA) delivering 187 L ha^-1^ at 269 kPa. The herbicide was applied with 20 g L^-1^ ammonium sulfate. The plants were assessed visually relative to the non-treated control 21 d after treatment (21 DAT) using a scale of 0 to 100, where 0 = no visible injury and 100% = complete desiccation. The number of survivors was recorded. Survivors from glufosinate treatment were grown to produce seed. Data were analyzed using hierarchal clustering in JMP Pro v. 12.

### Herbicide resistance profiling of a selected *A*. *palmeri* accession

Data from the differential tolerance evaluation were used to select an accession for further study. Accession 08-Lee-C had the most number of survivors with minimum injury. Seeds from 08-Lee-C and SS accessions were planted as described in the previous section. Seedlings (7.5–9 cm tall) were treated with the recommended dose of fomesafen (264 g ha^-1^) (Flexstar^®^, Syngenta), glyphosate (870 g ha^-1^) (Roundup PowerMAX^®^, Monsanto), dicamba (280 g ha^-1^) (Clarity^®^, BASF), and ALS inhibitors pyrithiobac (73 g ha^-1^) (Staple LX^®^, DuPont) and trifloxysulfuron (8 g ha^-1^) (Envoke^®^, Syngenta). The ALS inhibitors were applied with 0.25% non-ionic surfactant (Induce^®^, Helena Chemical Co., Collierville, TN, USA), respectively. Herbicide treatments were applied as described in the previous section. Mortality was evaluated 21 d after treatment. The experiment was conducted in a randomized complete block design as in the previous section. Data were analyzed using ANOVA in JMP Pro v. 12.

### Evaluation of tolerance level to glufosinate

A dose-response bioassay was conducted in the greenhouse to determine the tolerance level of 08-Lee-C and C1 to glufosinate. Seeds were sown in 15-cm diameter pots filled with commercial potting soil. Seedlings, 7.5-cm tall, were sprayed with 11 doses of glufosinate using a laboratory sprayer as described in the previous section. The 08-Lee-C and C1 accessions were sprayed with glufosinate at 0.0012 to 0.5940 kg ai ha^-1^; the SS accession was sprayed at 0.0006 to 0.5950 kg ha^-1^. Non-treated checks were included for each accession. The herbicide was applied with 20 g L^-1^ ammonium sulfate. Shoot biomass was harvested 21 DAT, dried at 60°C for 72 h, and weighed. The experiment was conducted in a randomized complete block design with four replications. Five plants were used per replication (20 plants total) per herbicide concentration.

Data were analyzed using SAS JMP Pro v. 13 in conjunction with SigmaPlot v.13 (Systat Software, Inc., San Jose, CA, USA) for nonlinear regression analysis. The percentage biomass reduction was fitted to a nonlinear, sigmoid, four-parameter logistic regression model defined by

y=c+[(d−c)/(1+e{−a(x−b)})]

where *y* represents the biomass reduction expressed as percentage relative to the non-treated control, *a* is the growth rate, *b* is the inflection point, *c* is the lower asymptote, *d* is the upper asymptote, and *x* is the glufosinate dose. The herbicide doses that would cause 50% growth reduction (GR_50_) were estimated using the fitted regression equation.

### Ammonia accumulation assay

To identify S and T plants without glufosinate treatment, a leaf disc assay was conducted to measure ammonia accumulation caused by the inhibition of photorespiration by glufosinate [40]. The assay was conducted using 50 non-treated plants each from 08-Lee-C and the C1 progeny. In addition, leaf tissues from three 08-Lee-C plants that were controlled (S) and three C1 plants that survived (T) glufosinate application at the whole plant level were also tested. From each plant, two leaf discs (5-mm diameter) were excised from the youngest, fully expanded leaf of 6.4-cm tall seedlings. One leaf disc was placed per well in a microtiter plate containing 200 μM glufosinate. The plate was sealed with micropore tape and placed on a bench under light for 24 h. The plate was moved to a -80 °C freezer to stop the reaction. After two freeze-thaw cycles, ammonia content was measured in a spectrophotometer (Pharma Spec UV-1700, Shimadzu, Columbia, MD) at 630 nm using a modified method by Molin and Khan [41]. Leaf discs from S plants were expected to have higher ammonia content than those from T plants.

### Glutamine synthetase (*GS2*) relative copy number

Leaf tissues were harvested from confirmed S and T plants (three each) without glufosinate treatment and stored at -80°C until processing. Leaf tissues were harvested also from plants treated with glufosinate, 24 HAT. Upon evaluation of plant response 21 DAT, leaf tissues from three S and T plants were used also to determine the relative copy number of *GS2* gene. Genomic DNA was extracted using the modified hexadecyltrimethylammonium bromide (CTAB) method [42]. Quantitative real-time PCR (qPCR) was used to determine the genomic copy number of *GS2* relative to a housekeeping RNA dead box helicase gene *GS2* in *A*. *palmeri*. The primer pair GS2-F (5’- ATACGGAGAAGGAAGGCAAAG -3’) and GS2-R (5’- TGTGGGTTCCCAAAGTAGTG-3’) were designed to amplify a region of the chloroplast *GS*. RNA dead box helicase gene primers A36-F (5’- TTGGAACTGTCAGAGCAACC-3’) and A36-R (5’-GAACCCACTTCCACCAAAAC-3’) were used as internal primers to normalize the samples for any differences in DNA quantities. Reactions were conducted in three technical replicates, and a negative control consisting of primer pairs with no template was included. An 8-fold serial dilution of genomic DNA samples, ranging from 0.00064 to 50 ng, was used to construct a standard curve. The slope of the standard curve was used to determine amplification efficiency (*E*). The qPCR reaction efficiency was 97% with an *R*^*2*^ of 0.9907 and a slope of 3.271 indicating good assay validation. Genomic DNA templates (2 ng) were amplified in a 25-μL reaction containing 12.5-μL Bio-Rad iQ SYBR Green Supermix, 2-μL of primers (1:1 mix of forward and reverse primers), and nuclease-free water. Reaction conditions included 10 min incubation at 94°C, then 40 cycles of 94°C for 15 s and 60°C for 1 min, followed by a melt-curve analysis to confirm single PCR product amplification. Data were analyzed using CFX Manager software (v.1.5). Relative *GS2* copy number was calculated as *ΔCt* = (*Ct*, *A*36 − *Ct*, *GS*2) according to the method described by Gaines *et al* [43]. Increase in *GS2* copy number was expressed as 2^*ΔC*t^. Results were expressed as the fold increase in *GS2* copy number relative to *RNA dead box helicase*.

### RNA-Seq analysis

#### Sample preparation for RNA-Seq

This experiment used leaf tissues from non-treated and treated, confirmed S and T plants. These were the same plants used for ammonia assay and *GS2* copy number determination. Tissues were collected 24 h after glufosinate application for RNA extraction. This collection time was selected to capture herbicide stress adaptation genes and because maximum absorption of glufosinate occurs 24 HAT [28]. Treatments were designated as non-treated S (susceptible without treatment, SWO), non-treated tolerant (tolerant without treatment, TWO), treated susceptible (SWT), and treated tolerant (TWT) plants with three biological replicates. Total RNA was extracted from young leaf tissues of S and T plants using PureLink RNA Mini Kit (Life Technologies, Carlsbad, CA, USA) following the manufacturer’s protocol. The extracted RNA was treated with DNase (Invitrogen, Carlsbad, CA, USA) to remove potential genomic DNA contamination, according to the manufacturer’s instructions. The samples were then sent to the Clemson University Genomics and Computational Biology Laboratory, South Carolina for sequencing the transcriptome.

#### Transcriptome sequencing and assembly

Total RNA was normalized, and converted to cDNA using the TruSeq RNA library kit v2.0 (Illumina). Final sequencing products were validated for size on an Agilent Bioanalyzer 2100 (Agilent Technologies, Waldbronn, Germany) and sequenced using a 2x125bp paired-end sequencing module on an Illumina HiSeq 2500 (Illumina). Raw sequence reads were assessed for quality using the FastQC software package (http://www.bioinformatics.babraham.ac.uk/projects/fastqc/) and preprocessed to remove sequence adapters and low quality bases with the Trimmomatic software [44]. A reference unigene assembly comprehensive of developmental stage, tissue source, and experimental conditions was prepared by concatenating all preprocessed reads and assembling with the Trinity software package [45]. The resulting unigene assembly was filtered for genuine coding sequences (e.g., sequences without internal stop codons or chimeras) with the TransDecoder software, and clustered by identity with the CD-HIT software [46] in an attempt to collapse homologs, but not paralogs, at high identity thresholds.

#### Differential gene expression

Paired-end reads from each individual were aligned to the *de novo* transcriptome using the Subread package [47, 48]. Samtools was used to convert alignments from sam to bam format, sort, and index [49, 50]. Subread’s featureCounts counted the number of reads that aligned to each gene in the transcriptome [47, 48]. The final gene counts were loaded into Bioconductor’s edgeR package for statistical analysis [51–55]. Variance between samples was visualized by a multidimensional scaling (MDS) plot. Volcano plots were generated for each comparison of samples. The criteria for differential gene expression included a fold-change ≥2 between compared groups and statistical significance at *P*≤0.05 [56]. Expression differences were compared between non-treated T and non-treated S (TWO vs SWO), treated T and treated S (TWT vs SWT), treated T and non-treated T (TWT vs TWO), and between treated S and non-treated S (SWT vs SWO).

#### Transcriptome annotation

The final reference assembly was annotated by blastx and blastp alignment to the non-redundant protein database at NCBI, the UniProt-swissprot database, and the uni-ref database to determine homology to known genes. Protein domains were determined by alignment to the HMMER database (http://hmmer.janelia.org). Signal peptides were determined with the SignalP software [57] and transmembrane regions predicted with tmHMM (http://www.cbs.dtu.dk/index.shtml). Gene ontology terms were derived from the best BLAST match [58] and clustered to determine enrichment using the Agbase tool [59].

The entire dataset was submitted as NCBI BioProject (PRJNA390774), which is a part of the U.S. National Library of Medicine of the National Institutes of Health. The 12 samples that were used to construct the transcriptome, and to run the differential gene expression comparisons were submitted as 12 separate BioSamples, SAMN07260017-SAMN07260028. The trimmed, paired-end .fastq files for each of the 12 samples were submitted to the Sequence Read Archive, SRR5759376—SRR5759387. Finally, the transcriptome was submitted to the Transcriptome Shotgun Assembly Sequence Database. The transcriptome, consisting of 72,780 transcripts, is under TSA submission: SUB2788796.

#### Sequence analysis of the glutamine synthetase 2 (*GS2*) gene

Glutamine synthetase 2 (*GS2*) gene sequences of the T and S biotypes were extracted and assembled from the transcriptome data. A 1296-bp *GS2* gene (431 amino acids) from S and T plants was sequenced. The full length *GS2* sequences of S and T plants were aligned using Sequencher 5.4.6 and BioEdit software packages to identify amino acid substitutions. Sequence alignment also included *GS2* sequences of other *Amaranthus* species (*A*. *viridis*, *A*. *albus*, *A*. *spinosus*, *A*. *hybridus*, *A*. *lividus* and *A*. *thunbergii*) available in the database at http://www.weedscience.org.

#### Heat map analysis

Differentially expressed genes associated with abiotic stress response were subsampled and subjected to heat map analysis. Normalized read count averages were calculated to produce biological expression profiles followed by hierarchical clustering to recursively merge expression based on pair-wise distances between non-treated T (TWO), non-treated S (SWO), glufosinate-treated T (TWT), and glufosinate-treated S (SWT) samples. Digital expressions were visualized between rows for normalized read count numbers (minimum & maximum) expression patterns. The expression pattern was generated using GENE-E tool (http://www.broadinstitute.org/cancer/software/GENE-E/).

#### Selection of candidate non-target genes

Genes that were commonly expressed between treated S and T, and between treated T and non-treated T plants were selected for further evaluation based on their gene ontologies (GO). Genes that were assigned with GO molecular function and biological process related to metabolism and signaling pathways (oxidoreductase activity, nuclear acid binding transcription factor activity, hydrolase activity, transferase activity, transmembrane transporter activity, transferase activity, protein transporter activity, biosynthetic process, small molecule metabolic process, signal transduction, homeostatic process, immune system process, cell wall organization, secondary metabolic process, nitrogen cycle metabolic process) were evaluated based on UniProt and their fold change. Contig assemblies that were consistently upregulated in the treated T (relative to treated S and non-treated T) with a significant P-value in the DESeq analysis were selected, for a total of 49 contigs. A subset of this list was generated based on known gene function. Contigs with predicted annotations related to stress response, signaling, transcription factors, and herbicide metabolism were selected as potential candidate NTS genes involved in glufosinate tolerance.

### Candidate gene validation by qRT-PCR

Two *A*. *palmeri* populations were treated with glufosinate at 0.37 kg ha^-1^ using the previously described procedure. Leaf tissues were collected three days before and 24 h after herbicide treatment. Tolerant and susceptible plants from each population were identified three weeks after herbicide treatment based on level of injury. Three biological replicates from the non-treated and treated samples from each biotype within each population were used for the validation experiment. Total RNA was extracted from leaf tissues using PureLink RNA Mini kit (Life Technologies, Carlsbad, CA). Genomic DNA was removed using DNAse I (Thermo Scientific, Waltham, MA). cDNA was generated from 5 μg total RNA using Reverse Transcription System first-strand cDNA synthesis kit (Promega). A 4-fold serial dilution of cDNA samples (1:1, 1:5, 1:25, 1:125) was used to construct a standard curve. Seven of the candidate NTS genes were subjected to real-time quantitative PCR with primers designed using Primer3 tool. Two genes (β-tubulin, RNA helicase) were used as internal controls for normalization of gene expression. Primers had an amplification efficiency of 96 to 110%.

The expression level of 7 candidate NTS genes was measured in 24 plants. Quantitative real-time PCR reactions were conducted in a 12-μL volume containing 6.25 μL of SyberGreen Master Mix, 1 μL of 1:25 diluted cDNA, and 0.5 μL of 10 μM primers (1:1 mix of forward and reverse primers). Amplification was performed in a Bio-Rad MiniOpticon System PCR machine (CFX96, Bio-Rad Laboratories, Inc., Hercules, CA, USA) under the following conditions: 10 min at 94 °C, 40 cycles of 94 °C for 15 s and 60 °C for 1 min, followed by a melt-curve analysis to confirm single PCR product amplification. Data were analyzed using CFX Manager software (v.1.5). Slopes for target and internal control genes were equivalent as observed in amplification plots. Comparative CT method was used to calculate relative expression levels as 2^−ΔΔCt^ where ΔCT = [CT target gene–geometric mean (CT internal control genes)] and ΔΔCT = [ΔCt tolerant − ΔCt susceptible]. Wilcoxon non-parametric test (α = 0.05) was used to determine statistical difference in gene expression between tolerant and susceptible biotypes.

## Results

### Differential response of *A*. *palmeri* accessions to glufosinate

All field populations represented by the accessions tested were susceptible to glufosinate. The majority were killed 100% by 0.55 kg ha^-1^ glufosinate, except for some, which had few survivors. The 120 accessions had differential levels of susceptibility to glufosinate, separating into three groups based on mortality and levels of injury of survivors (Table 1). The first group was composed of the 88 most sensitive accessions. The second group, composed of 28 accessions, were had 94 to 99% mortality with survivors incurring 60–99% injury. The third group consisted of four segregating accessions with 88 to 97% mortality. Survivors from these accessions incurred 30–80% injury. Of the possibly segregating accessions, only survivors from 08-Lee-C were able to produce sufficient seeds to conduct further experiments. Seven percent of 08-Lee-C survived glufosinate treatment, of which 4% of treated plants had <61% injury and produced seeds. Survivors from other recalcitrant accessions were not able to produce enough seeds due to either having high injury (>75%) or low number of survivors, which were all males. Considering that plants growing in the field tend to be more robust than those in the greenhouse, the likelihood of escapes in the field may be higher than that in the greenhouse. Plants in the field also do not receive uniform amounts of herbicide for various reasons such as partial coverage by other plants or differential plant size. In addition, plants maintained in the greenhouse that are well-watered and cultured under warm temperatures grow faster and reach the recommended spraying heights earlier than those growing in the field due to less variation in ambient conditions [60].

**Table 1:** Cluster analysis of *A*. *palmeri* accessions treated with glufosinate at 0.55 kg ha^-1^.

Cluster	Number of accessions	Mortality (%)			Mean frequency of plants at different levels of injury (%)					
		Mean	Min	Max	0–10% injury	11–30% injury	31–60% injury	61–80% injury	81–99% injury	100% injury
1	88	100	100	100	0	0	0	0	0	100
2	28	98	94	99	0	0	1	1	0	98
3	4	92	88	97	0	1	3	4	0	92

### Response of 08-Lee-C to other foliar herbicides

The 08-Lee-C accession was susceptible to dicamba and fomesafen, but resistant to glyphosate and ALS inhibitors, which are commonly used herbicides. The mortality of 08-Lee-C was 98% and 99% with fomesafen and dicamba, respectively (Table 2). On the other hand, 08-Lee-C was controlled poorly with glyphosate (EPSPS inhibitor) (61%) as well as with ALS inhibitors trifloxysulfuron (25%), and pyrithiobac (21%). This accession is resistant to these two commonly used modes of action, thus limiting the herbicide options for post-emergence weed mitigation.

**Table 2:** Response of *A*. *palmeri* (08-Lee-C) to foliar-applied herbicides.

Herbicide	Mortality (%)^a^	Mode of action^b^
**Dicamba**	99	Synthetic auxin
**Fomesafen**	98	PPO inhibitor
**Glufosinate**	93	Glutamine synthetase inhibitor
**Glyphosate**	61	EPSP synthase inhibitor
**Pyrithiobac**	21	ALS inhibitor
**Trifloxysulfuron**	25	ALS inhibitor

^a^Uniform-sized plants (7.5–9 cm tall) were sprayed with dicamba (280 g ha^-1^), fomesafen (264 g ha^-1^) glufosinate (0.55 kg ha^-1^), glyphosate (870 g ha^-1^), pyrithiobac (73 g ha^-1^), and trifloxysulfuron (8 g ha^-1^). Mortality was recorded 21 d after herbicide application.

^b^PPO- protoporphyrinogen oxidase, EPSP- enolpyruvyl shikimate-3-phosphate, ALS- acetolactate synthase

### Resistance level to glufosinate

**Fig 1:**
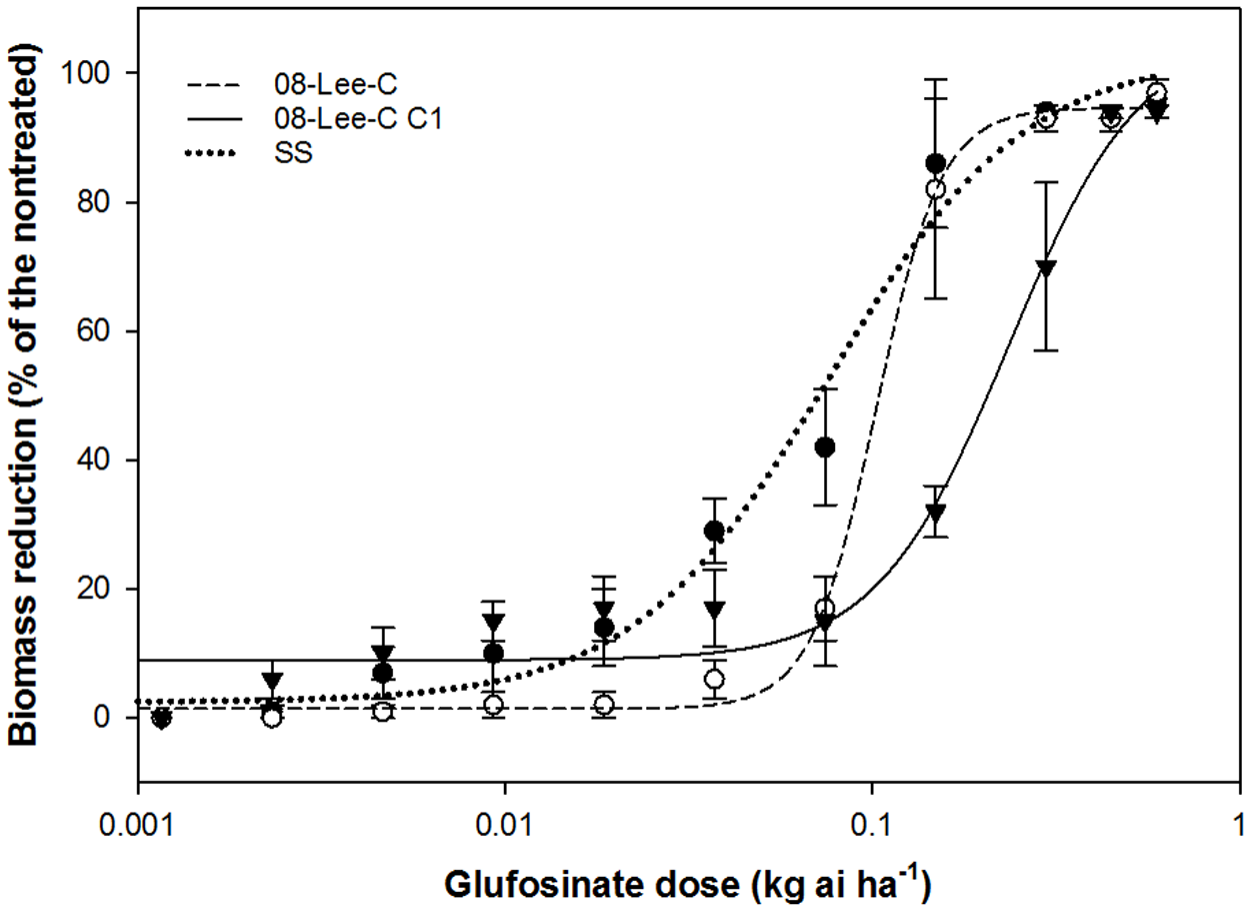
Shoot biomass reduction (%) of 08-Lee-C, C1, and SS *A*. *palmeri* accessions, 21 days after glufosinate treatment. Treatment means were plotted with a regression curve. Data were best described with nonlinear, sigmoidal, four-parameter logistic regression function.

The response of *A*. *palmeri* to 11 doses of glufosinate fitted a sigmoidal, logistic function (Fig 1). The glufosinate doses required to reduce growth by 50% (GR_50_) were 0.076, 0.110, and 0.214 kg ha^-1^ for SS, 08-Lee-C, and C1 accessions, respectively (Table 3). Based on these GR_50_ values, the level of tolerance to glufosinate in 08-Lee-C and and C1 accessions was 1.4- and 2.8-fold, respectively, relative to the susceptible standard (SS). The GR_50_ increased 2-fold, from 110 g ha^-1^ in 08-Lee-C to 214 g glufosinate ha^-1^ in C1.

**Table 3:** Glufosinate dose required to reduce growth by 50% (GR_50_) in *A*. *palmeri* 08-Lee-C, C1 and SS accessions.

Accession	GR_50_	Confidence Intervals^a^	T/S^b^
	kg ai ha^-1^		
**08-Lee-C**	0.110	0.097–0.123	1.44
**C1**	0.214	0.184–0.244	2.80
**SS**^c^	0.076	0.064–0.088	

^a^ 95% confidence intervals.

^b^ Tolerance levels (T/S) calculated using the GR_50_ of the tolerant accession relative to the susceptible standard.

^c^Herbicide-susceptible standard accession.

### Ammonia accumulation in response to glufosinate

**Fig 2:**
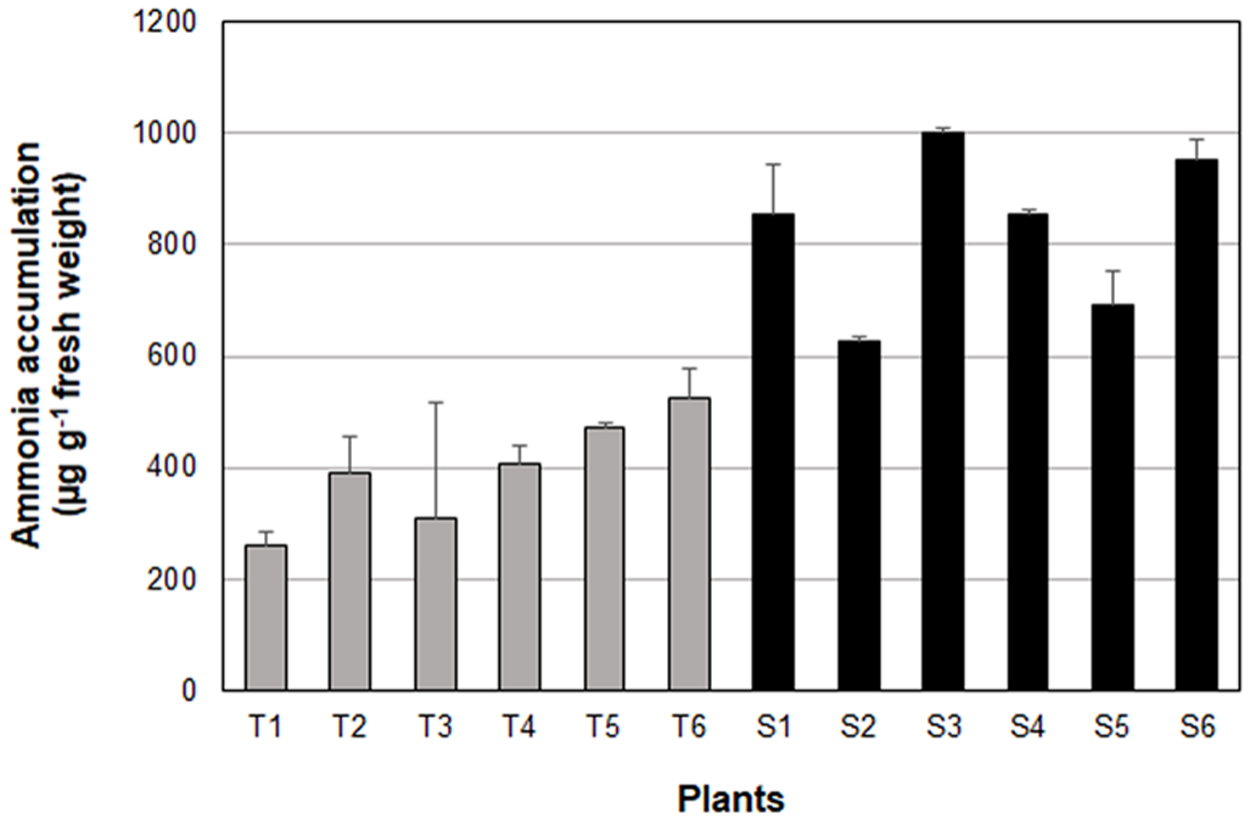
Ammonia content in glufosinate-tolerant (T) and –susceptible (S) *A*. *palmeri*. Error bars represent standard error. White bars = S plants; gray bars = T plants.

Glutamine synthetase, the target site of glufosinate, is a nuclear-coded enzyme that catalyzes the conversion of L-glutamate to L-glutamine by assimilating ammonia in the cytoplasm and plastids, but predominantly in the chloroplast of green tissues [21]. Ammonia accumulation is a direct response to the inhibition of this pathway by glufosinate. The ammonia concentration in S plants was 830 μg g^-1^ fresh leaf tissue (±60) and was 394 μg g^-1^ fresh weight (±40) in T plants (Fig 2). The S plants accumulated 2X more ammonia than the T plants, indicating rapid depletion of functional glutamine synthetase as a consequence of glufosinate treatment.

### Glutamine synthetase 2 (*GS2*) relative copy number

**Fig 3:**
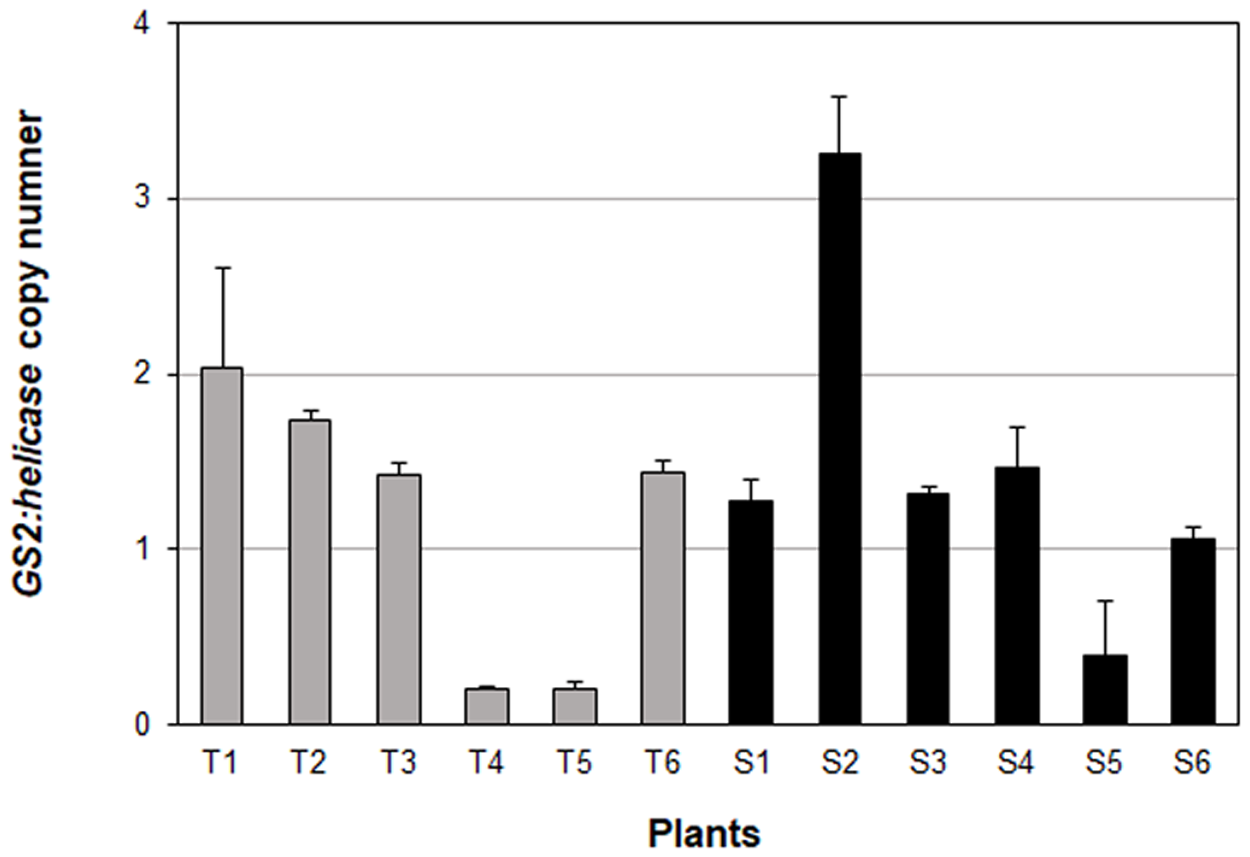
Relative copy number of *A*. *palmeri* GS2 in glufosinate-susceptible (S) and -tolerant (T) plants. Error bars represent standard deviation of the mean. Gray bars = T plants; black bars = T plant.

The relative *GS2* copy number of S and T plants ranged from 1 to 3 (Fig 3). Similar *GS2* copies were detected in both S and T plants, indicating that differential tolerance to glufosinate is not due to amplification of the *GS2* gene. Transcriptome analysis also revealed that *GS2* was not differentially expressed between T and S plants, which indicated that differential tolerance to glufosinate in *A*. *palmeri* was not due to changes in expression of the target enzyme.

### Glutamine synthetase gene sequence analysis

**Fig 4:**
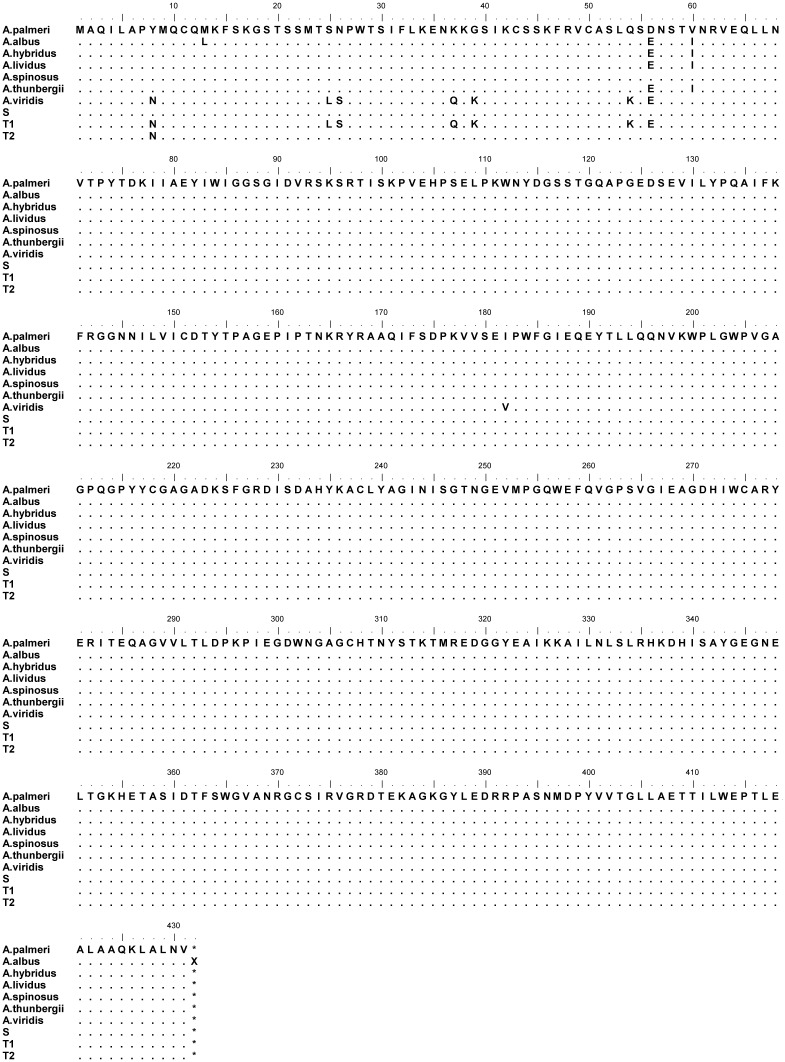
Multiple alignment of the plastidic glutamine synthetase (GS2) amino acid sequences in *Amaranthus*. A. palmeri (reference *A*. *palmeri*), T1 and T2 = GS2 alleles of glufosinate-tolerant *A*. *palmeri* biotype, S = GS2 sequence of glufosinate-susceptible *A*. *palmeri* biotype.

Glutamine synthetase plays a primary role in plant nitrogen metabolism by catalyzing the conversion of glutamate to glutamine [21]. Glutamine synthetase in higher plants exists in two major isoforms: GS1 in the cytosol and GS2 in the chloroplast/plastids [61]. The cytosolic form (GS1) is the predominant isoform in roots and non-green tissues [62]. The chloroplast form of glutamine synthetase (GS2) is the major isoform in leaves, which is primarily responsible for recycling ammonia during photorespiration and synthesis of glutamine [63]. In our study, two different alleles of *GS2* were observed in the T plants and one in the S plants. The nucleotide sequences obtained from the two biotypes had 97–99% identity with *GS2* sequences from other *Amaranthus* species (*A*. *albus*, *A*. *hybridus*, *A*. *spinosus*, *A*. *lividus*, *A*. *thunbergii*, *and A*. *viridis*). The T biotype differed in six amino acids in the upstream region of *GS2* when compared to the S biotype. Seven nonsynonymous point mutations (Tyr_8_Asn, Ser_25_Leu, Asn_26_Ser, Lys_37_Gln, Gly_39_Lys, Gln_54_Lys, Asp_56_Glu) in the upstream region were detected in one of the *GS2* alleles of the T biotype (Fig 4). The second allele of the T biotype harbored only the Tyr_8_Asn substitution. These nonsynonymous substitutions identified in the *A*. *palmeri* T biotype also occur in herbicide-susceptible *A*. *viridis*, indicating that these substitutions do not contribute to tolerance to glufosinate. Other nucleotide polymorphisms between T and S plants were synonymous mutations.

### Global transcriptional changes in *A*. *palmeri* 24 h after glufosinate application

**Fig 5:**
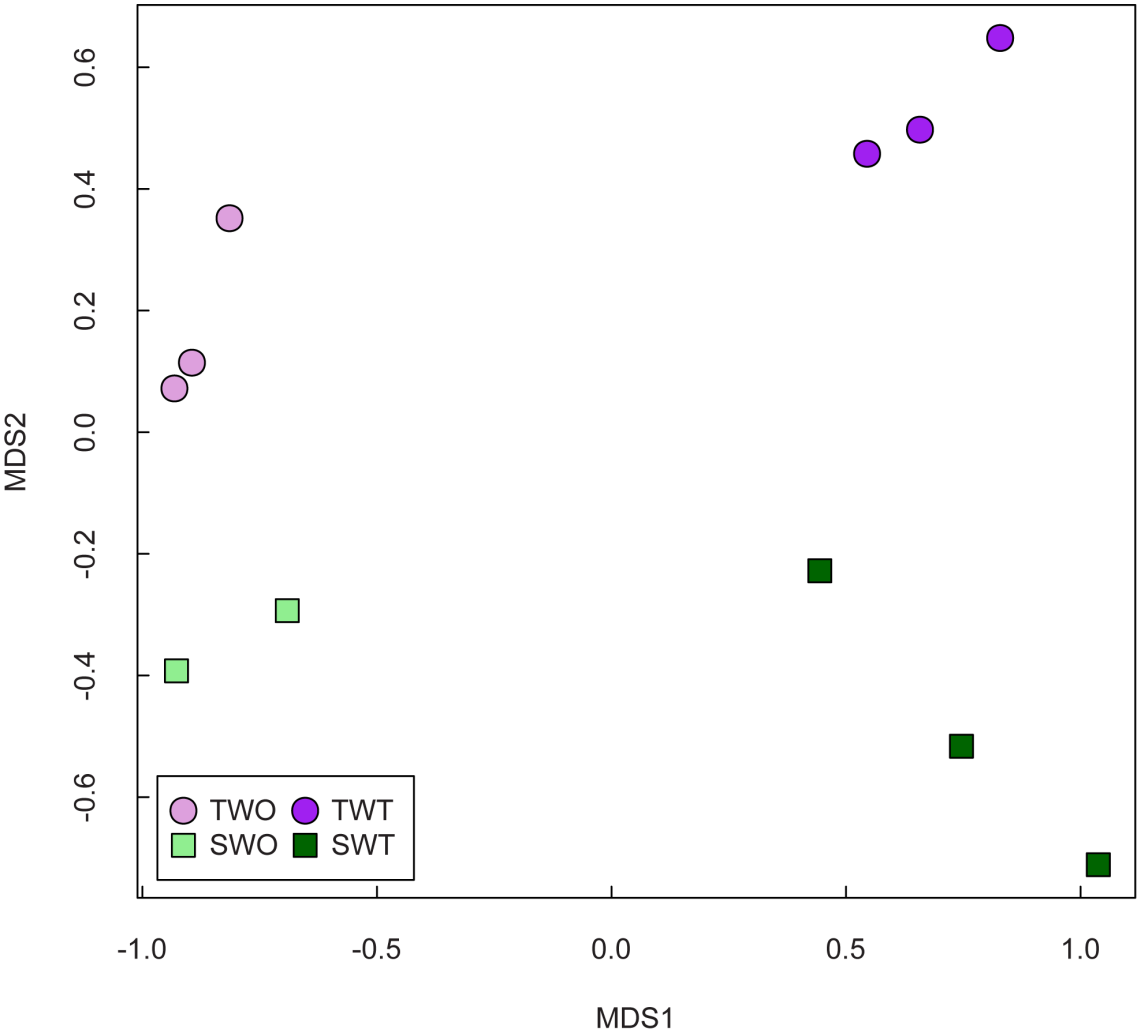
Multidimensional scaling (MDS) plot showing the relationship between sample types. TWO = non-treated tolerant, TWT = treated tolerant, SWO = non-treated susceptible, SWT = treated susceptible.

A reference cDNA transcriptome consisting of 72,794 sequences was assembled (Table 4). Treatment samples were similar as indicated in the multidimensional scaling (MDS) plot (Fig 5). Biological replicates from the same treatment clustered together indicating low bias and variation among treatment samples. One or more GO terms were assigned to 33,516 sequences with 76,455 GO assignments in total for biological process (31.9%), cellular component (10.8%) and molecular function (57.3%) categories.

**Table 4:** Summary of statistics for transcriptome assembly.

	Reads (n)	Bases (Mb)	Average length (bp)
**Illumina raw reads**	1,667,277, 670	8409.7	125
**Assembled contigs**	72,780	49.15	675
**Annotated sequences (blastX)**	65,282	-	-
**Sequences assigned with GO terms**	33,294	-	-

#### Background differences between susceptible and tolerant plants

Pairwise comparison between non-treated S and T plants showed 438 differentially expressed genes, 158 of which were downregulated and 280 were upregulated in the T plants relative to S plants (Table 5). Genes that were notably more expressed in the T plants relative to the S plants without herbicide treatment included cytochrome P450s (*Cyp72A219*, *Cyp86b*, *Cyt77A*, *Cyt71A*, *Cyt76A*, *Cyt86A*), transporters (ABC transporter), transferases (glycosyltransferase, acylytransferases), antioxidants (glutathione-S-transferase, superoxide dismutase), and genes related to lipid metabolism (esterase lipase).

**Table 5:** Differentially expressed genes putatively involved in differential tolerance to glufosinate in *Amaranthus palmeri*.

Level of gene expression	Number of differentially expressed genes^a^							
	TWO vs SWO		SWT vs SWO		TWT vs TWO		TWT vs SWT	
	Repressed	Induced	Repressed	Induced	Repressed	Induced	Repressed	Induced
**>1–2**	2	0	589	353	1277	1048	0	0
**>2–3**	43	26	1228	994	1846	1229	33	11
**>3–4**	58	72	662	677	756	679	94	65
**>4–5**	27	64	328	389	299	351	74	41
**>5–6**	13	36	109	218	125	182	63	31
**>6–7**	4	22	72	146	55	135	23	22
**>7–10**	8	51	45	175	37	113	47	33
**>10**	3	9	10	39	1	21	23	7
**Total**	158	280	3043	2991	4396	3758	357	210
	438		6034		8254		567	

^a^TWO vs SWO: non-treated tolerant (T) relative to non-treated susceptible (S) plants; SWT vs SWO: treated S relative to non-treated S plants;

TWT vs TWO: treated T relative to non-treated T plants; TWT vs SWT: treated T relative to treated S plants

#### Genes induced by glufosinate application

**Fig 6:**
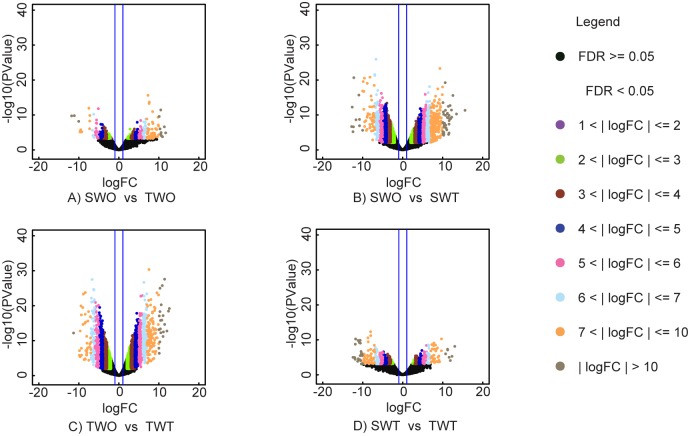
Volcano plots depicting differential gene expression between treatments. A) Treated susceptible (S) relative to non-treated S plants (SWT vs SWO), B) Treated tolerant (T) relative to treated S plants (TWT vs SWT), C) treated T relative to non-treated T plants (TWT vs TWO), and D) treated T plants relative to non-treated S plants (TWO vs SWO). The x-axis shows the log fold change or relative abundance. The *P* value (-log base 10) for differential gene expression is plotted on the y axis. Dots in black represent genes that did not achieve significant changes in expression; colored dots on the left indicate genes with significantly downregulated expression and colored dots on the right indicates genes with significantly upregulated expression.

**Fig 7:**
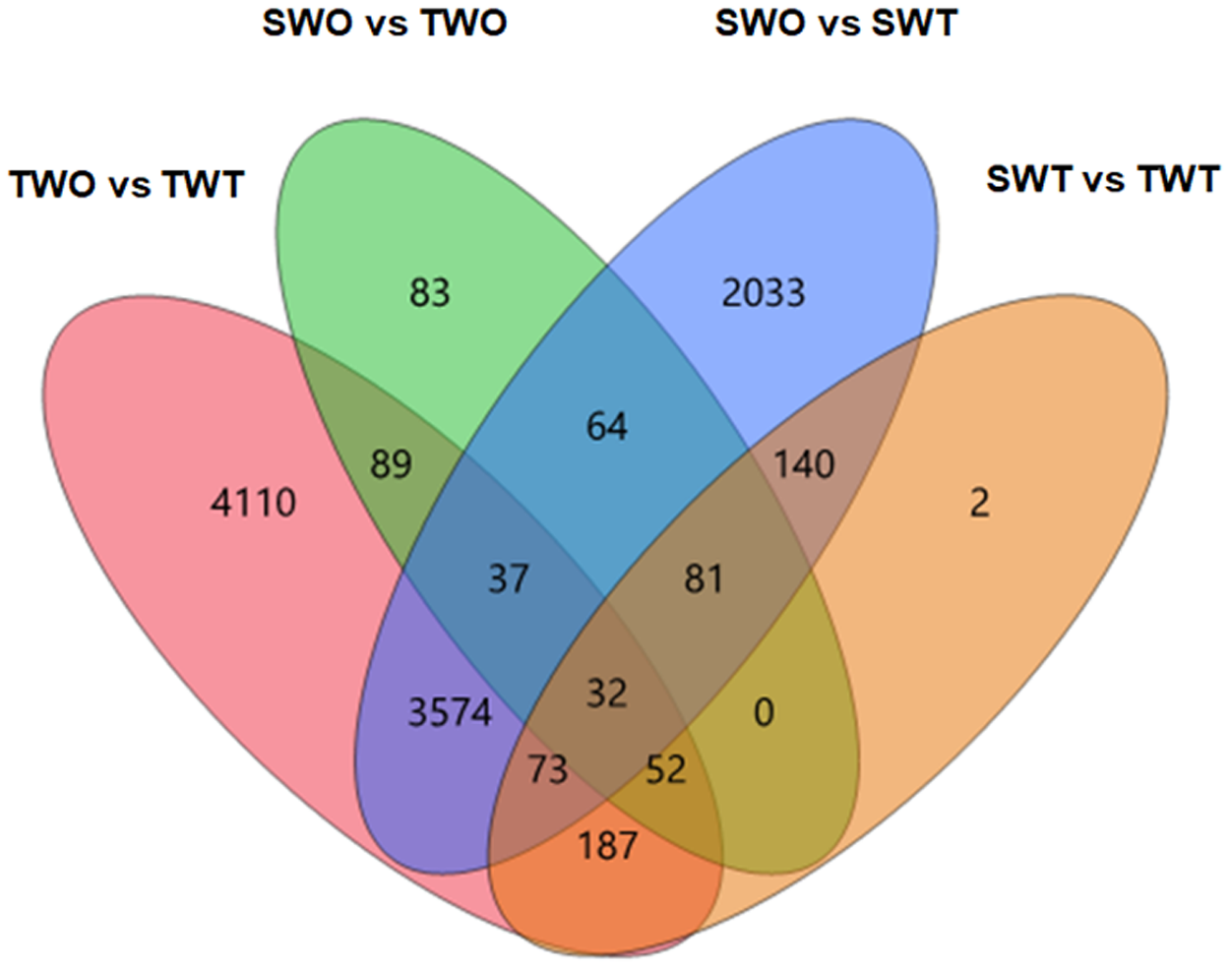
The number of differentially expressed genes common or specific to treated and non-treated T and S plants. A 4-way Venn diagram depicting the distribution of differentially expressed genes across all pairwise comparisons. The number within each shaded area is the number of differentially expressed genes common in each compared treatments.

**Fig 8:**
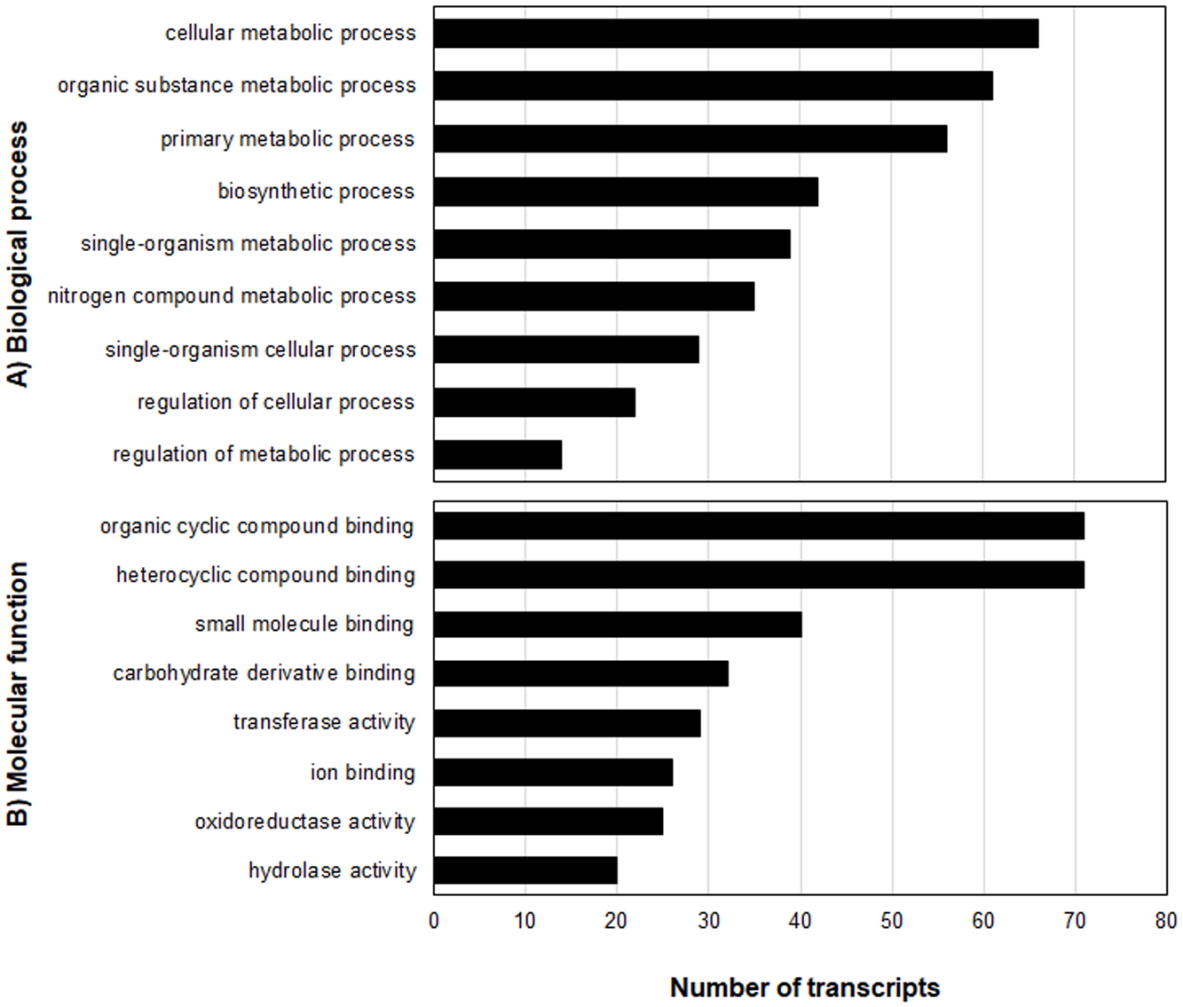
Biological processes (A) and molecular functions (B) of upregulated genes in treated T relative to treated S plants.

**Fig 9:**
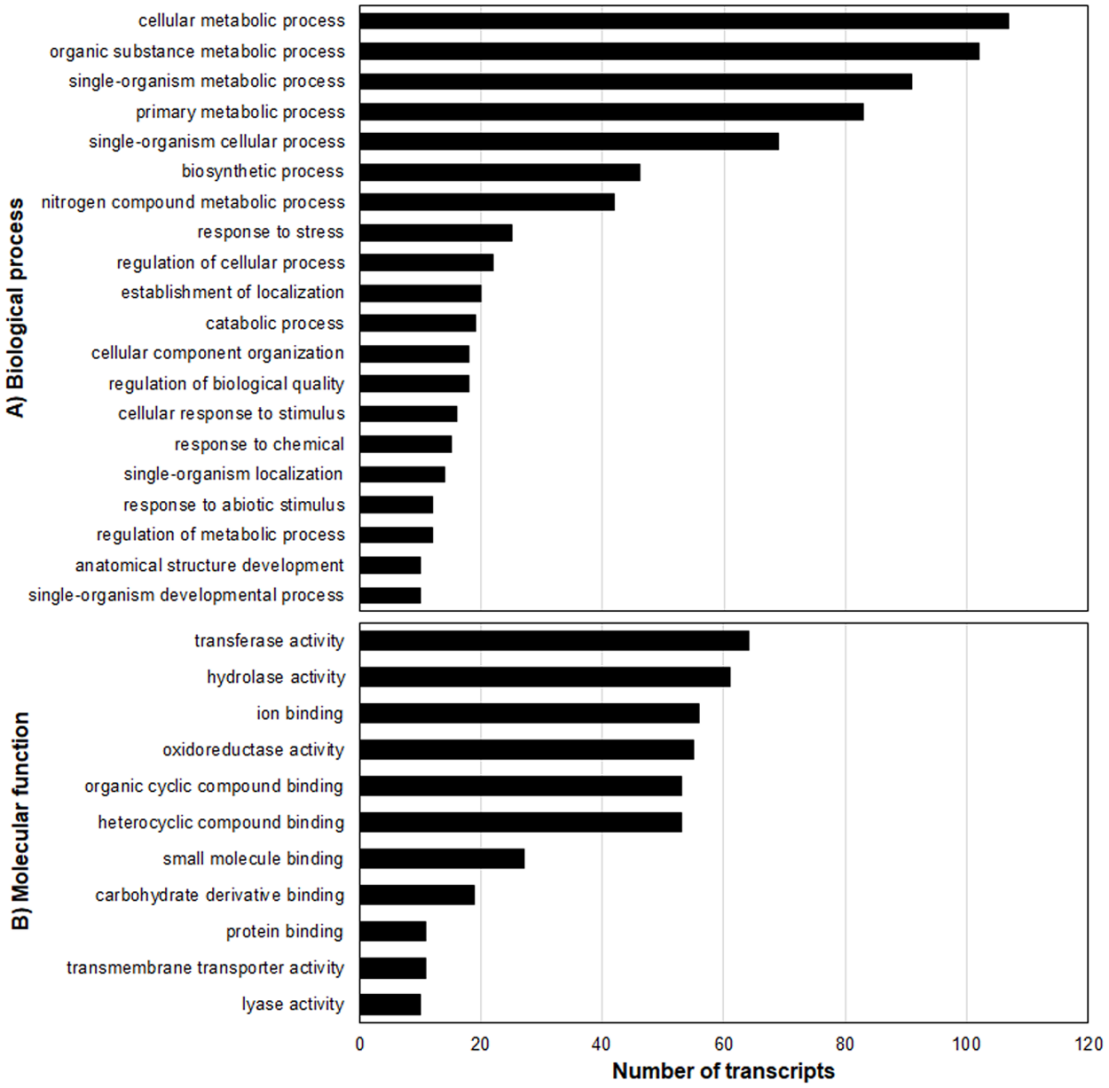
Biological processes (A) and molecular functions (B) of downregulated genes in treated T relative to treated S plants.

Relative to the respective non-treated checks, 8154 genes were affected by glufosinate application in the T plants and 6034 genes in the S plants (Table 5, Figs 6 and 7). Comparison between treated T and S plants revealed 567 genes that were more repressed or more induced by glufosinate in the treated T plants. Overall, there were 210 upregulated and 357 downregulated genes in the treated T relative to the treated S plants (Figs 8 and 9). One hundred-five glufosinate-responsive genes were differentially expressed in both treated T (32 genes) and S plants (73 genes) (Fig 7). In addition, 239 genes that were differentially expressed between treated (52 genes) and non-treated (187 genes) T plants were more highly repressed or more induced in treated T plants than in treated S plants. Of these 239 genes, the majority were related to biosynthetic process, cellular nitrogen compound and small molecule metabolic processes, response to stress, and oxidoreductase activity (Fig 9). Among the upregulated genes of this 239- gene subset, 91 were induced by glufosinate in T plants, including genes putatively annotated as NAC transcription factor, *CYP94a1*, and ABC transporter b. The majority of upregulated genes that were differentially expressed between treated T and S plants, and that were also differentially expressed relative to their respective non-treated counterparts, were related to nitrogen compound metabolic processes, oxidoreductase activity, nucleotide binding, and transferase activity. Two genes that were repressed exclusively in the treated T plants relative to the treated S plants were folypolyglutamate synthase and caffeic acid 3-o-methyltransferase (Fig 7).

**Fig 10:**
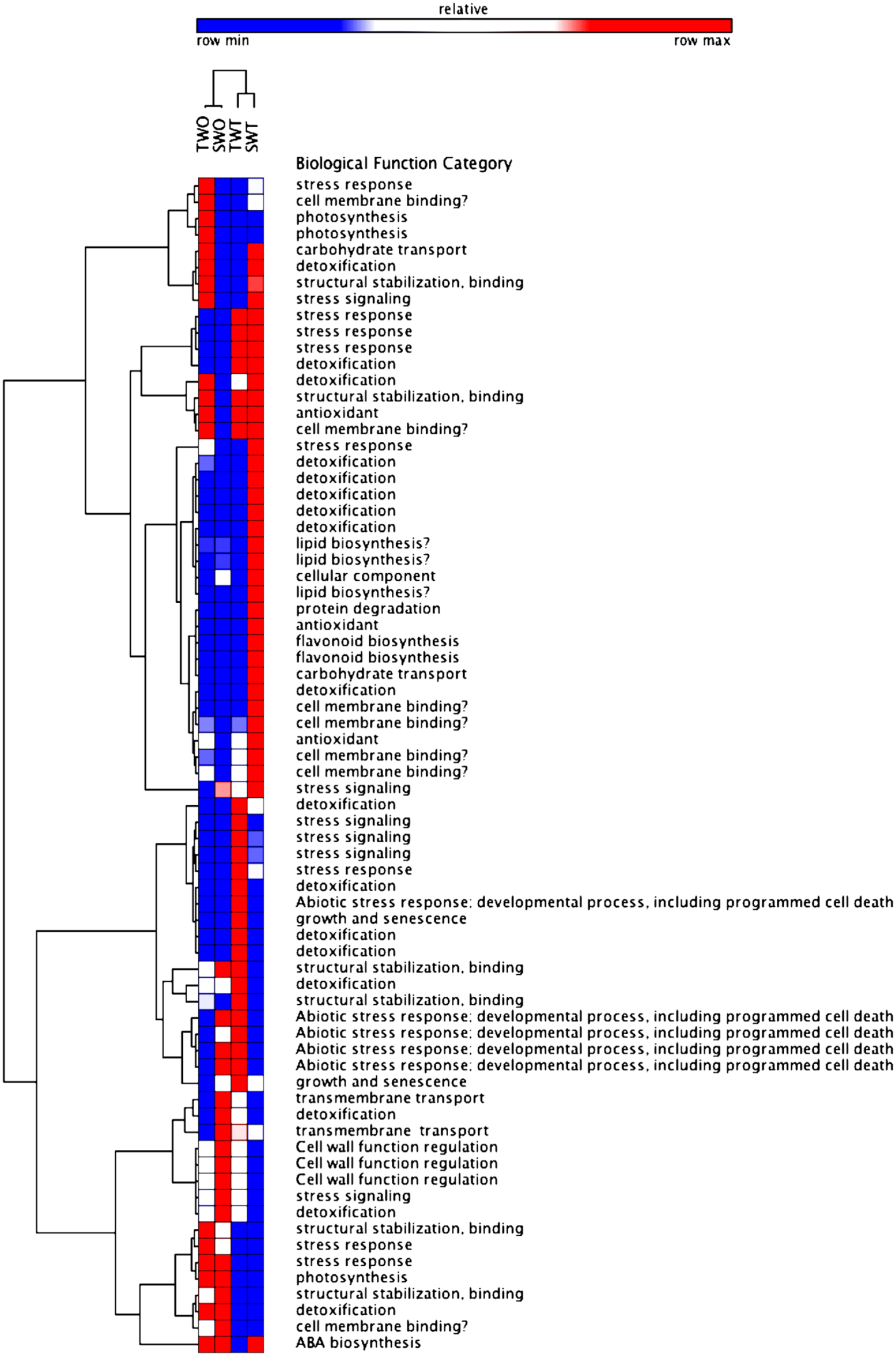
Heat map analysis of genes that are putatively related to abiotic stress response in *A*. *palmeri*. TWO (non-treated tolerant), SWO (non-treated susceptible), TWT (glufosinate-treated tolerant), SWT (glufosinate-treated susceptible).

The functional classification of selected differentially expressed genes (DEGs) associated with stress response and herbicide metabolism was examined to investigate the pattern of transcriptome regulation that occurred during glufosinate treatment (Fig 10). Glufosinate treatment triggered the expression of genes related to stress response and xenobiotic detoxification as expected. Some genes associated with photosynthesis, structural stabilization, cell membrane binding, stress response, and detoxification were repressed. Increased expression of non-target site (NTS) genes related to stress response, stress signaling, detoxification, abiotic response, cell structure stabilization, and growth and senescence was observed in treated T plants. Genes that were exclusively induced in treated T plants were annotated as coding for transmembrane protein 45b, heat stress transcription factor B, hypersensitive-induced response protein, cytochrome P450 (*Cyp72A219*, *94A2*, *Cyt86b1*-like), transcription factor, ethylene-responsive transcription factor, glutathione S-transferase (*GST*), zinc finger protein *constans-like 10*, and *NAC* transcription factor (Fig 8, Tables 5 and 6). These candidate genes likely play a role in the adaptation of *A*. *palmeri* to glufosinate and, possibly, also to other herbicides.

**Table 6:** Upregulated genes in glufosinate-treated and non-treated tolerant (T) plants, and in glufosinate-treated T relative to treated susceptible (S) plants, assigned with Gene Ontology molecular function and biological process related to metabolism and signaling pathways.

GO function	Contig	Gene annotation	Fold change^a^	
			TWT_n_/TWO_n_	TWT_n_/SWT_n_
**biosynthetic process**	Pa27529	50S ribosomal protein chloroplastic	**8.47**	**9.02**
**biosynthetic process**	Pa29824	60s ribosomal protein l13a-2	**7.60**	6.25
**biosynthetic process**	Pa38623	phenazine biosynthesis-like domain-containing protein 1 isoform x2	**6.93**	3.92
**biosynthetic process, cellular nitrogen compound metabolic process, nucleic acid binding transcription factor activity**	Pa65724	cyclic dof factor 1-like	**3.67**	4.46
**biosynthetic process, small molecule metabolic process**	Pa35601	phosphoribosylaminoimidazole chloroplastic-like	**9.24**	11.22
**cellular amino acid metabolic process, biosynthetic process, small molecule metabolic process**	Pa17844	shikimate chloroplastic	**2.49**	4.41
**cellular amino acid metabolic process, secondary metabolic process**	Pa39917	3-isopropylmalate dehydratase -like protein	**4.86**	5.79
**cellular amino acid metabolic process, small molecule metabolic process**	Pa60555	probable low-specificity l-threonine aldolase 1	**7.04**	5.40
**cellular nitrogen compound metabolic process**	Pa52820	putative polyprotein	**2.55**	4.88
**cellular nitrogen compound metabolic process**	Pa63676	CTP synthase	**1.64**	3.18
**cellular nitrogen compound metabolic process**	Pa71553	gag-pol polyprotein	**5.93**	7.09
**cellular nitrogen compound metabolic process**	Pa8879	zinc finger bed domain-containing protein ricesleeper 1-like	**7.63**	9.05
**cellular nitrogen compound metabolic process, biosynthetic process, signal transduction**	Pa63868	two-component response regulator arr9 isoform x1	**2.20**	3.43
**cellular nitrogen compound metabolic process, biosynthetic process**	Pa37812	NAC transcription factor	**8.25**	7.80
**cellular nitrogen compound metabolic process, biosynthetic process, signal transduction**	Pa51700	two-component response regulator arr5-like isoform x2	**1.62**	2.58
**cellular nitrogen compound metabolic process, response to stress, biosynthetic process**	Pa37809	heat stress transcription factor b-2b-like	**3.01**	2.88
**cellular nitrogen compound metabolic process, response to stress, biosynthetic process, signal transduction**	Pa13900	RNA polymerase ii c-terminal domain phosphatase-like 1	**3.52**	3.83
**hydrolase activity, acting on glycosyl bonds**	Pa26833	PREDICTED: alpha-glucosidase-like [*Beta vulgaris* subsp. vulgaris]	**6.20**	4.47
**hydrolase activity, acting on glycosyl bonds**	Pa42036	alkaline neutral invertase cinv2-like	**3.40**	3.15
**hydrolase activity, acting on glycosyl bonds, response to stress**	Pa69030	beta-amylase chloroplastic	**3.94**	5.41
**nucleic acid binding transcription factor activity, biosynthetic process, signal transduction**	Pa49594	auxin-responsive protein iaa29	**4.11**	4.51
**nucleic acid binding transcription factor activity, cellular nitrogen compound metabolic process, biosynthetic process**	Pa47424	NAC transcription factor 25-like	**1.98**	3.34
**nucleic acid binding transcription factor activity, cellular nitrogen compound metabolic process, response to stress, biosynthetic process, signal transduction**	Pa38292	ethylene-responsive transcription factor abr1	**5.24**	4.92
**oxidoreductase activity**	Pa10467	cytochrome P450 cyp72A219-like	**8.56**	4.42
**oxidoreductase activity**	Pa40402	internal alternative NAD H-ubiquinone oxidoreductase mitochondrial	**5.22**	3.49
**oxidoreductase activity**	Pa45867	nitronate monooxygenase	**6.26**	4.28
**oxidoreductase activity**	Pa51578	-dopa dioxygenase extradiol-like protein	**6.44**	5.06
**oxidoreductase activity**	Pa56011	short-chain type dehydrogenase reductase-like	**7.89**	3.99
**oxidoreductase activity**	Pa60473	cytochrome P450 94a1-like	**2.90**	3.34
**oxidoreductase activity, cellular amino acid & metabolic process, cellular amino acid metabolic process, homeostatic process, oxidoreductase activity**	Pa10326	5 -adenylylsulfate reductase chloroplastic- partial	**3.47**	4.04
**oxidoreductase activity, small molecule metabolic process**	Pa44392	abscisic acid 8 -hydroxylase 2	4.15	4.07
**response to stress, immune system response**	Pa52955	macpf domain-containing protein at1g14780	1.80	3.090
**response to stress, immune system response**	Pa62900	heat shock protein 83	4.42	4.39
**response to stress, signal transduction, immune system process**	Pa42133	chitin elicitor receptor kinase 1-like	3.35	3.35
**response to stress, signal transduction, immune system process**	Pa69811	receptor-like protein kinase at3g47110	3.40	6.46
**response to stress, transport, transmembrane transport**	Pa53135	mitochondrial phosphate carrier protein mitochondrial-like	11.42	4.21
**signal transduction**	Pa60381	receptor-like serine threonine-protein kinase sd1-8 isoform x1	5.53	3.69
**signal transduction**	Pa63442	PREDICTED: uncharacterized protein LOC104887975	3.32	5.56
**transferase activity, transferring acyl groups**	Pa67068	uncharacterized acetyltransferase at3g50280-like	10.27	4.38
**transferase activity, transferring alkyl or aryl (other than methyl) groups**	Pa19271	glutathione s-transferase-like protein	10.47	6.70
**transferase activity, transferring glycosyl groups, biosynthetic process**	Pa49933	7-deoxyloganetin glucosyltransferase-like	6.04	4.24
**transferase activity, transferring glycosyl groups, response to stress, bioysnthetic process, small molecule metabolic process, cell wall organization or biogenesis**	Pa57353	gdp-l-galactose phosphorylase 2-like	5.46	5.23
**transmembrane transporter activity**	Pa14919	peroxisomal nicotinamide adenine dinucleotide carrier-like	1.68	4.46
**transmembrane transporter activity**	Pa21499	calcium-transporting atpase plasma membrane-type	4.46	3.36
**transmembrane transporter activity**	Pa35784	mate efflux family protein 9-like	3.46	3.26
**transmembrane transporter activity**	Pa63432	anoctamin-like protein at1g73020	3.79	3.22
**transmembrane transporter activity, cellular nitrogen compound metabolic process, transport, small molecule metabolic process**	Pa63215	ABC transporter b family member 2-like	4.66	6.57
**transport**	Pa60553	outer envelope protein mitochondrial	3.15	7.27

^a^TWT = treated T plants; TWO = non-treated T plants; SWT = treated S plants

**Fig 11:**
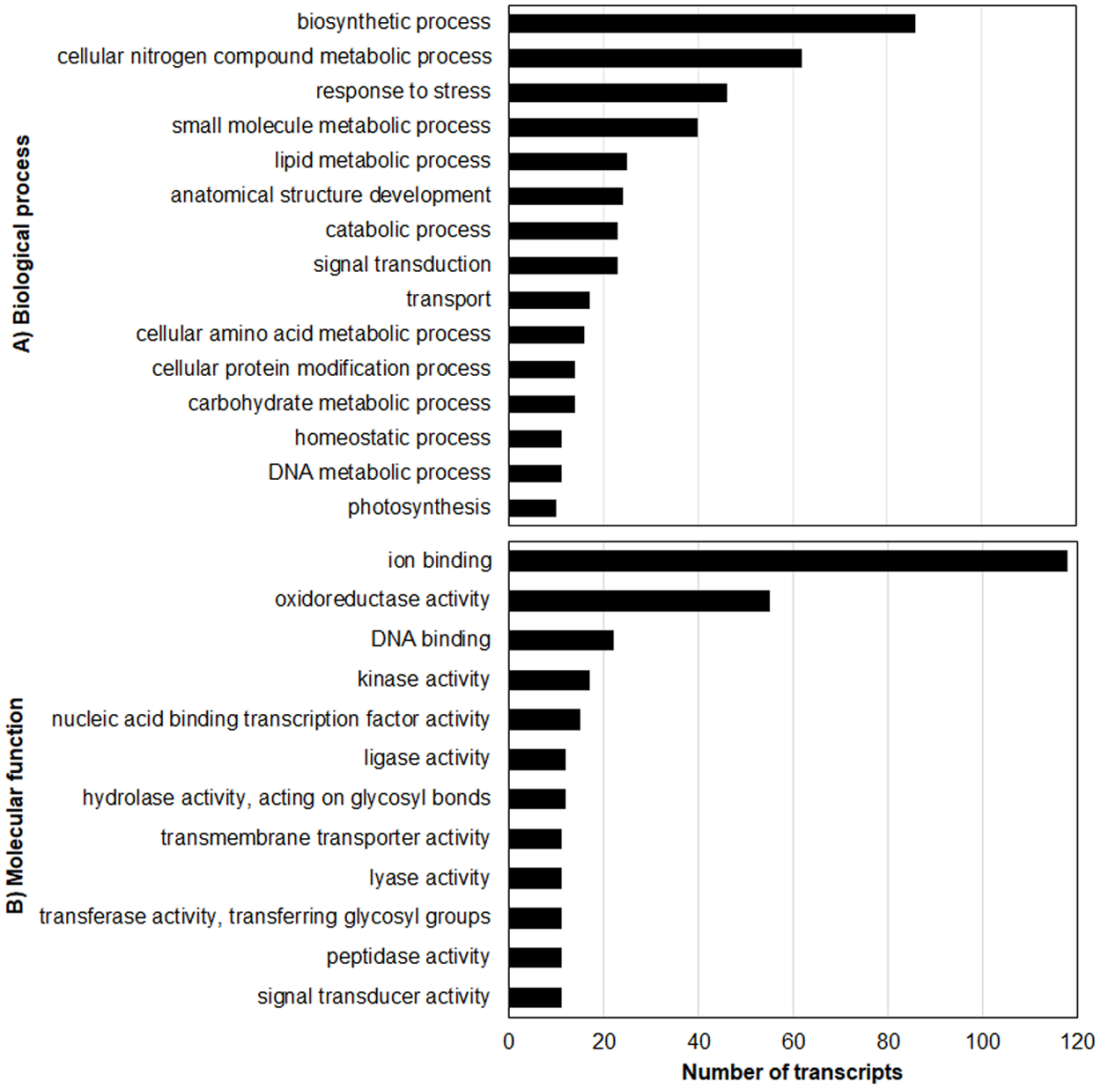
Biological processes (A) and molecular functions (B) of differentially expressed genes that are common in treated T relative to treated S and non-treated T plants.

Of the 239 genes that were more highly induced or repressed in the treated T plants, 49 were consistently upregulated. These consistently upregulated genes are involved in biosynthetic process, cellular nitrogen compound metabolic process, nucleic acid binding transcription factor activity, oxidoreductase activity, stress response, signal transduction, transferase activity, transmembrane transporter activity, and transport (Table 6 and Fig 11). A subset of 13 glufosinate-inducible genes which are related to detoxification, stress signaling, and transport are candidate genes involved in conferring some tolerance to glufosinate. These include ABA 8’-hydroxylase, ABC transporter, chitin elicitor receptor kinase, cytochrome P450 72A, cytochrome P450 94A, GST, heat stress transcription factor, heat shock protein 83, ethylene response transcription factor, NAC transcription factor, NAC transcription factor 25, NADH ubiquinone oxidoreductase, and nitronate monooxygenase (NMO) (Table 7). These genes were induced >2-fold in treated T plants relative to treated S plants and non-treated T plants.

**Table 7:** Candidate non-target genes, identified by RNA-Seq analysis, that are potentially involved in conferring differential tolerance to glufosinate in *A*. *palmeri*.

Contig	Gene annotation	Fold change^a^		Function
		TWT_n_/TWO_n_	TWT_n_/SWT_n_	
**19271**	Glutathione S-transferase (*GST*)	10.47	6.7	Detoxification
**10467**	Cytochrome P450 CYP72A219	8.55	4.42	Heme-thiolate monoxygenase; detoxification
**37812**	NAC transcription factor	8.24	7.8	Transcription regulator in plant stress response
**38292**	Ethylene-response transcription factor abr1	5.24	4.91	ABA signaling pathway in response to stress response
**40402**	NAD H-ubiquinone oxidoreductase	5.21	3.49	Detoxification
**63215**	ABC transporter b family member 2	4.66	6.56	Transmembrane transport
**62900**	Heat shock protein 83	4.31	8.2	Molecular chaperone; stress signaling
**44392**	ABA 8’-hydroxylase	4.15	4.07	ABA catabolism
**45867**	Nitronate monooxygenase (*NMO*)	6.26	4.27	Detoxification
**42133**	Chitin elicitor receptor kinase 1 (*CERK1*)	3.35	3.35	Cell surface receptor toward biotic and abiotic stresses
**37809**	Heat stress transcription factor b	3.01	2.87	Transcription regulator for heat shock proteins; stress signaling
**60473**	Cytochrome P450 94a1	2.9	3.33	Detoxification
**47424**	NAC transcription factor 25-like	1.97	3.34	Abiotic stress response

^a^TWT = treated tolerant plants; TWO = non-treated tolerant plants; SWT = treated sensitive plants

### Validation of selected genes using qRT-PCR

**Fig 12:**
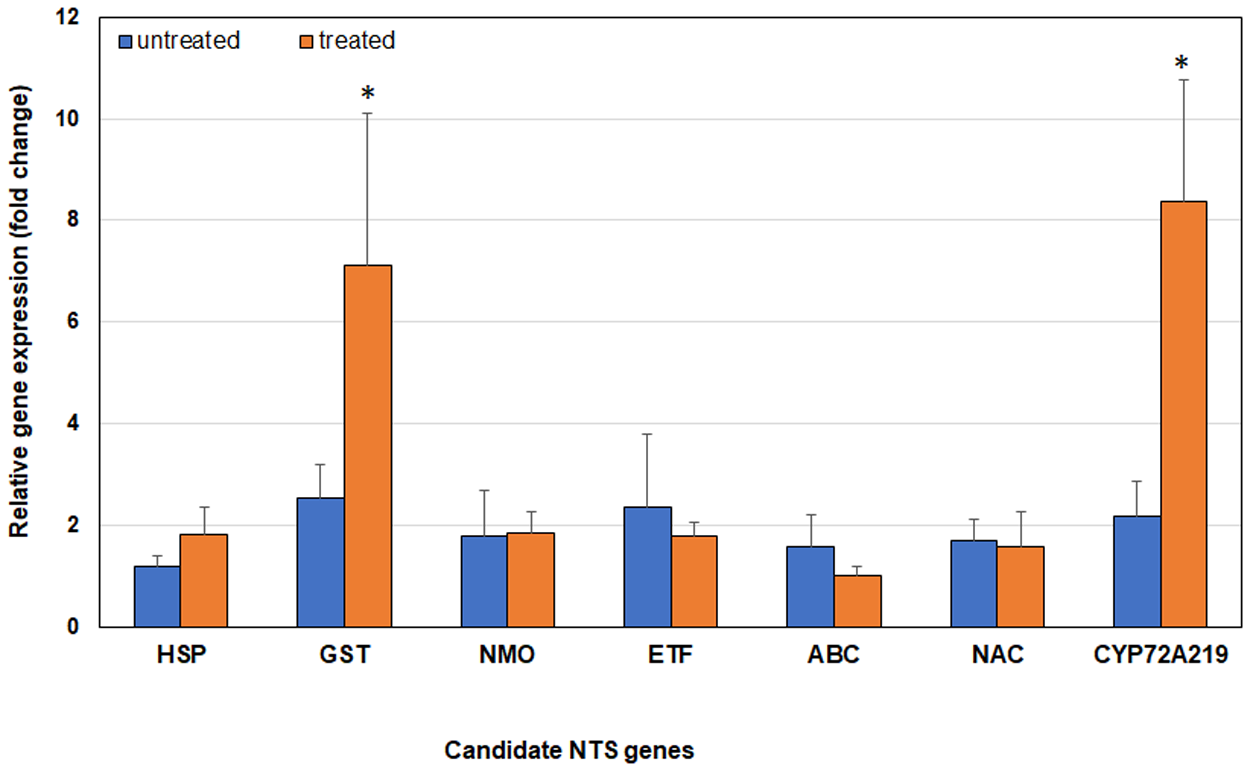
Gene expression fold-change of seven candidate NTS genes in glufosinate-tolerant relative to –susceptible *Amaranthus palmeri*. CYP72A219 was highly expressed in treated tolerant plants. GST was more induced in only one of the treated tolerant plants relative to susceptible ones. Untreated = before glufosinate treatment, Treated = 24 h after glufosinate treatment. Error bars represent standard error. HSP = heat shock protein, GST = glutathione S-transferase, NMO = nitronate monooxygenase, ETF = ethylene-responsive transcription factor, ABC = ABC transporter, NAC = NAC transcription factor, CYP72A219 = cytochrome P450 CYP72A219. Wilcoxon non-parametric test was used to compare differential gene expression between tolerant and susceptible biotypes. An asterisk denotes significant difference at P<0.05.

The expression of 7 candidate NTSR genes was measured in two *A*. *palmeri* accessions using quantitative real-time PCR. Gene expression was similar in the non-treated tolerant and susceptible plants. The 7 genes were induced in T and S plants upon glufosinate treatment. Five genes (*HSP*, *NMO*, *ETF*, *ABC*, *NAC*) were not differentially expressed between treated tolerant and treated susceptible plants. *GST* was differentially expressed in only one of the treated tolerant plants. On the other hand, *CYP72A219* was expressed eight times higher in all the treated tolerant plants relative to the susceptible ones (Fig 12).

## Discussion

### Differential tolerance to glufosinate among *A*. *palmeri* accessions

The differential response to glufosinate among 120 *Amaranthus palmeri* accessions demonstrates variation in herbicide efficacy on a weed species (Table 1). Several factors affect glufosinate activity even on a single species; these include temperature, light, relative humidity, time of day, plant size/age, and dose [30, 64–69]. Variation in environmental conditions was minimal in the greenhouse. Variation in plant factors was minimized by maintaining seedlings of the same size. The impact of time of day on glufosinate activity was eliminated by applying the herbicide at about the same time in each repetition of the experiment. Accessions with a few survivors reflect some heterogeneity within the population, as expected of this highly diverse species. Projecting such diversity to field conditions, where all the factors mentioned above can vary, we expect to see a higher frequency of individuals that would escape weed control activities in the field. Many such escapes could have been subjected to sub-lethal doses as a consequence of plant, environmental, and application variables. Controlling these escaped individuals, or adopting a weed management strategy that controls escaping genotypes, is a critical step in mitigating the accumulation of non-target-site genes that could eventually endow population-level resistance to herbicides [70].

### Resistance profiling of the 08-Lee-C recalcitrant accession

Herbicides impose strong abiotic stresses to weeds in crop fields, managed turfgrass, gardens, roadsides, and other chemically managed areas. The evolution of resistance in populations of weedy species is an increasing problem worldwide. The recalcitrant *A*. *palmeri* accession, 08-Lee-C, was resistant to glyphosate and ALS-inhibiting herbicides, trifloxysulfuron and pyrithiobac (Table 2). The occurrence of resistance to multiple herbicides in 08-Lee-C is not surprising because this field was sprayed with glyphosate and ALS inhibitors for several years. This field was planted with glyphosate-tolerant crops for more than three years and had been exposed to ALS inhibitors in the years prior when the grower was planting conventional soybean. Resistance to glyphosate and ALS inhibitors among *A*. *palmeri* in Arkansas is widespread [71]. Fomesafen, a PPO herbicide with soil and foliar activity, was first commercialized in the 1960s and had been used by farmers mainly for soybean. The usage of fomesafen, and almost all other herbicides in soybean, dropped when glyphosate-tolerant soybean was introduced in the mid-1990s. Upon the explosion of glyphosate-resistant *Amaranthus* species, soybean farmers reverted to using fomesafen and its use was expanded to cotton to control glyphosate-resistant *Amaranthus* species. The farmer of this field, like many others, had been using only glyphosate to control weeds. Although glufosinate had not been used in this field, some *A*. *palmeri* individuals had higher tolerance to glufosinate, allowing them to survive a single application. This population was susceptible to the field use rate of glufosinate, with a few individuals that would tend to escape treatment. Being an obligate outcrossing species, *A*. *palmeri* exhibits high genetic diversity, which facilitates its tendency to evolve herbicide resistance. Intensive use of glufosinate in this field, in a manner that allows escapes to produce seed, will accelerate the evolution of resistance through accumulation of multiple low-impact tolerance genes as demonstrated already in some species, including *Lolium rigidum* [72] and *A*. *palmeri* [73]. Tolerance traits can accumulate and get fixed in the population as selection pressure continues.

### Ammonia accumulation in response to glufosinate

Ammonia accumulation is directly related to glufosinate toxicity. Inhibition of glutamine synthetase and ammonia accumulation triggers a cascade of reactions, including inhibition of ribulose-1,5-bisphosphate carboxylase/oxygenase (RUBISCO) enzyme [74] and photosystem electron flow [75], affecting photosynthesis [30, 76] leading to plant death. Ammonia reduces pH gradient across the membrane, which uncouples photophosphorylation [75]. Elevated levels of ammonia accumulated in glufosinate-treated rice and soybean cell cultures [77, 78]. In our study, glufosinate-sensitive plants accumulated 2X more ammonia than the T plants (Fig 2). Similarly, glufosinate-sensitive *L*. *perenne* ssp. *multiflorum* from Oregon accumulated 1.6X more ammonia than the resistant population [25]. Increased ammonia level in S plants after glusofinate treatment is the consequence of rapid depletion of functional glutamine synthetase. Reduced ammonia accumulation in T plants indicates the presence of mechanism(s) that reduce the impact of glufosinate on plant function. Such mechanisms could either be reduced binding affinity of glufosinate by target site modification or, mechanisms external to the herbicide- binding site (NTSM) including detoxification and others. The latter applies to the glufosinate-tolerant *A*. *palmeri* plants.

### Glutamine synthetase (*GS2*) copy number

Gene amplification conferring herbicide resistance has been identified in GR weeds such as *A*. *palmeri*, *A*. *tuberculatus*, *K*. *scoparia*, and *L*. *multiflorum* [43, 79, 80]. The GR plants contain multiple copies of *EPSPS*, the target site of glyphosate, which results in increased production of EPSPS enzyme allowing the plant to function normally despite the presence of glyphosate. This mechanism has not been observed with other herbicide target genes either because it is exclusive to the *EPSPS* regulatory process, or simply because it has not been investigated in other herbicide target genes. Amplification of *GS2*, in glufosinate-resistant weeds, is not yet reported. However, a 4- to 11-fold amplification of *GS2* in alfalfa cell culture lines resulted in increased GS enzyme production, endowing resistance to glufosinate [81]. In our study, S and T *A*. *palmeri* had similar copies (1–3) of *GS2* indicating that tolerance to glufosinate was not due to *GS2* amplification (Fig 3). This was supported by the fact that *GS2* transcripts were not different between S and T plants.

### Glutamine synthetase (*GS2*) gene sequence

A rare individual in a population may harbor a mutation in the herbicide-binding site that can alter the folding structure of the protein, resulting in reduced binding affinity of the herbicide. Resistance to ALS-inhibiting herbicides in weeds, in most cases, is due to mutation(s) in one or more of the binding domains in the ALS enzyme [82]. The higher frequency of SNPs in *GS2* of T plants could predispose such individuals in the population to accumulate nonsynonymous nucleotide substitutions. However, genetic polymorphisms may not always get translated to protein polymorphisms, and only certain amino acid mutations will result in herbicide resistance [82]. In the current study, most of the polymorphisms observed in the nucleotide sequence of *GS2* in T plants were synonymous. Although seven amino acid substitutions in the upstream region were detected in one of the alleles of the T biotype, these substitutions also occur in glufosinate-sensitive *A*. *viridis* and is in a region outside of the substrate-binding domain (Fig 4). A Tyr_8_Asn substitution was detected in the two GS2 alleles of the T biotype. Asparagine and tyrosine are both polar, uncharged amino acids, hence Tyr_8_Asn substitution may not alter the physiological and physicochemical stability of the plastid GS enzyme. The presence of this trivial amino acid substitution in glufosinate-tolerant plants as well as similar *GS2* copies as that of the S plants suggests that non-target-site tolerance factors are involved. Transcriptome analysis could inform us on differential tolerance mechanisms. A glufosinate-resistant *L*. *perenne* ssp. *multiflorum* from Oregon, USA, which showed similar level of ammonia accumulation to the tolerant *A*. *palmeri* in our study, harbors a Asn_171_Asp *m*utation (GAC TO AAC) in the *GS2* gene which confers resistance to glufosinate [25].

### Tolerance level to glufosinate in 08-Lee-C and C1

After one cycle of selection, the GR_50_ for C1 increased from 1.44-fold to 2.80-fold relative to SS, reflecting increased frequency of tolerant plants in C1 (Table 3). Although a low frequency (<10%) of the plants survived exposure to glufosinate, the increase in GR_50_ after one cycle of glufosinate selection is indicative that the population has become less sensitive to glufosinate after one cycle of purifying selection. Because glufosinate had not been used in the field, the frequency of survivors was low, and the tolerance level was only 1.44 to 2.80-fold, it is likely that these plant variants have low-level, non-target site resistance. We are possibly capturing an early phase of herbicide resistance evolution. Should the population continue to be under selection pressure from glufosinate, the probability of the population acquiring additional adaptive alleles and expressing resistance to field use rate of glufosinate would increase.

### Transcriptome of *A*. *palmeri* and candidate NTS genes involved in glufosinate tolerance

A reference cDNA transcriptome consisting of 72,794 sequences was assembled for *A*. *palmeri* (BioProject PRJNA390774, TaxId 107608). The transcriptome of *A*. *hypochondriacus* had 57,658 assembled sequences [36]. Our data demonstrated broad effects of glufosinate on several metabolic pathways, as expected of a herbicidal compound, including nitrogen assimilation and metabolism similar to what is reported in *Arabidopsis* [83]. One of the apparent functional categories to which glufosinate-responsive genes belong is protein families known to participate in metabolism, stress response, and defense, with the majority of these genes potentially associated with abiotic stress response signaling and chemical detoxification pathways (Figs 10 and 11). Stress response genes are inducible by many other herbicides or stress factors. Generally, abiotic stress such as herbicide, salinity, and drought modulates the expression of genes that are involved in signaling cascades and in transcriptional control [84], genes that code for proteins involved in membrane protection [85], and genes that are involved in water and ion uptake and transport [86, 87]. These stress-regulated genes are activated to counteract the stress effects, maintain homeostasis, and adapt. Cell membrane receptor-kinases, stress signaling genes, detoxification-related genes, and antioxidants were activated upon glufosinate treatment in both S and T plants (Fig 10). Peroxidase and superoxide dismutase, for example, were upregulated to help counteract the oxidative stress caused by lipid peroxidation resulting from glufosinate treatment. This indicates that plants undergo extensive transcriptional adjustment in response to herbicide-induced stress. Activation of herbicide-stress-response genes is hypothesized to be initiated by a herbicide sensor, which triggers the activation of regulator genes, which causes a cascade of reactions to either detoxify the herbicide or protect the plant from herbicide-mediated stress [35].

Two genes, folypolyglutamate synthase and caffeic acid 3-o-methyltransferase, were repressed in the treated T relative to S plants, but were not differentially expressed in other pairwise comparisons. Both are involved in one-carbon transfer and phenylpropanoid biosynthesis [88, 89]. The phenylpropanoid pathway serves as a rich source of metabolites in plants, especially for lignin biosynthesis and the production of flavonoids, coumarins, hydrocinnamic acid conjugate, cutins and lignins [90, 91]. Phenylpropanoids are involved in plant defense, structural support and survival [91, 92]. Repression of genes involved in phenylpropanoid biosynthesis indicate less allocation of carbon resources to these plant products in the T plants after glufosinate treatment compared to the S plants, indicating a shift in carbon allocation to other intermediates, which are more critical for survival under herbicide stress.

Thirteen candidate genes were identified which included ABA 8’-hydroxylase, ABC transporter (ABC), chitin elicitor receptor kinase (CERK1), cytochrome P450 72A (CYP72A219), cytochrome P450 94A, glutathione S-transferase (GST), heat stress transcription factor, NAC transcription factor, NAC transcriptor 25, ethylene-response transcription factor (ETF), heat shock protein 83 (HSP), NADH ubiquinone oxidoreductase and nitronate monooxygenase (NMO) (Table 6). The expression of these candidate genes was induced by glufosinate treatment. Delye [35] proposed a model of NTS resistance mechanism in which herbicide stress triggers the expression of ‘protectors’ and ‘regulators’, as well as epigenetic modifiers which enable the plant to survive herbicide stress. Protector genes include cytochrome P450, oxidase, peroxidases, esterases, hydrolases, glutathione S-transferases and transporters, which play roles in reducing the efficacy of herbicide by detoxification. ‘Regulator’ genes are involved in transcriptional, post–transcriptional, and post-transductional control such as transcription factors, micro-RNAs, and kinases [35]. It is noteworthy that the candidate NTS genes could act as either ‘protector’ or ‘regulator’ based on their functions. Cytochrome P450, GST, NADH ubiquinone oxidoreductase, and ABC transporter proteins have roles in pesticide detoxification [93–98]. Chitin elicitor receptor kinase, ABA 8’-hydroxylase, ethylene-response transcription factor, heat stress transcription factor, and NAC transcription factor are involved in stress response signaling and regulation [35, 89, 99–102].

Of the seven candidate genes subjected to qRT-PCR validation experiment, only two genes, (cytochrome P450 *CYP72A219* and *GST*) were associated with tolerance to glufosinate (Fig 12). The induction of *GST* and *CYP72A219* suggests that T plants are able to deactivate glufosinate to some extent. Induction of cytochrome P450 and GST indicates possible conversion of the herbicide into a less toxic metabolite. The biochemical role of cytochrome P450-mediated herbicide metabolism has been well established in herbicide-resistant weed species. Plant cytochrome P450s facilitate the detoxification of toxic xenobiotics by catalyzing oxygen- and NADPH-dependent mono-oxygenation reactions which convert herbicide into a more hydrophilic metabolite [103]. Hundreds of P450 genes exist in higher plants. For example, *Arabidopsis thaliana* and *Oryza sativa* possess 272 and 458 putative P450 genes, respectively [104]. RNA-Seq transcriptome analysis of *L*. *rigidum* identified CYP72A genes to be involved in metabolic resistance to diclofop [37]. Non-target-site ACCase and ALS resistance in *Alopecurus myosuroides* [95, 96], *Stellaria media* [105], *Lolium* [96, 106–108], *Sinapis arvensis* [109], *Echinochloa phyllopogon* [110], and *Digitaria sanguinalis* [111] were reported previously to be facilitated by cytochrome P450 enzymes. Upregulation of *CYP72A* and *CYP94A* was reported in a multiple-herbicide-resistant *E*. *phyllopogon* population [112]. Similarly, *CYP94A1*, a plant cytochrome P450-catalyzing fatty acid omega hydroxylase, was induced by chemical stress in *Vicia sativa* and by bentazon treatment in soybean [113, 114]. Some cytochrome P450 genes in the CYP71A family were also demonstrated to be involved directly in herbicide metabolism in crops, such as *O*. *sativa CYP71A31* and Z*ea mays CYP71A2*8 [115, 116].

The involvement of glutathione *S*-transferases (GSTs) in herbicide resistance is reported in several weed species. Glutathione *S*-transferases are ubiquitous enzymes that catalyze the conjugation of harmful xenobiotics to reduced glutathione, facilitating their metabolism, sequestration or removal [117]. The primary factor for atrazine selectivity in corn is the activity of a soluble GST, which detoxifies atrazine by forming an atrazine-glutathione conjugate [118]. In a recent transcriptome study, increased expression of *GST* is associated with diclofop resistance in *L*. *rigidum* and nicosulfuron tolerance in *Z*. *mays* [37, 116]. GST also functions as an antioxidant, protecting plants from herbicide-mediated oxidative stress by scavenging reactive oxygen species [119]. Increased expression of glutathione transferase gene (*AmGSTF1*) in multiple-resistant *A*. *myosuroides* led to accumulation of flavonoids which protects the plant from herbicide injury [120]. It has been reported that glutathione transferase orchestrate tolerance to abiotic stress through their ability to regulate redox signaling pathways that activate defense genes [121]. In glufosinate-tolerant *A*. *palmeri*, GST is possibly involved in converting glufosinate into a less toxic metabolite following possible minimal phase I detoxification by CYP72A219 as well as in protecting the plants against oxidative stress and lipid peroxidation from glufosinate phytoxicity.

Other candidate genes were identified by RNASeq, but were not differentially expressed in the validation experiment, including as the ABC transporter, NAC transcription factor, NMO, HSP, and ethylene transcription factor. Although these might not be involved in conferring some level of tolerance to glufosinate, their involvement in detoxification of toxic xenobiotic compounds and in stress response have been reported. For example, NMO in *A*. *thaliana* is associated with detoxification of the allelochemical benzoxazolin [122]. Increased expression of NADH ubiquinone oxidoreductase and induction of P450 genes were involved in resistance to pyriproxyfen insecticide in *Bemisia tabaci* [123]. Induction of heat shock proteins is associated with drought and oxidative stress in *Brassica juncea* [124] and *A*. *thaliana* [125], respectively. Plant ABC transporters have been associated with the movement of herbicide conjugates [126, 127]. Modifications of ABC transporters have been suspected in some cases of weed resistance to glyphosate or paraquat [128, 129]. Tolerance of *Arabidopsis thaliana* to paraquat is endowed by a mutation in the plasma membrane-localized ABC transporter, which resulted in reduced herbicide uptake in plant cells [130]. NAC transcription factors and ethylene-response transcription factors play an important role in the regulation of the transcriptional reprogramming associated with plant stress response such as cold, drought, and salinity [131–134].

Diversity in gene expression and regulation is an important factor driving herbicide resistance evolution [135]. As gene expression regulation also involves post-transcriptional and post-translational controls, protein expression of the identified genes may need to be investigated. Because of genetic diversity, plants have the potential to overcome herbicide stress through a concerted action of multiple genes. Plants with low-level tolerance showed greater induction of abiotic stress-protection- and detoxification-related genes than S plants. Thus, survival from glufosinate treatment is facilitated by stress-protection/stress-adaptation genes. Differential expression of stress-protection genes in a population can enable some individuals to survive herbicide application. Tolerance-related genes can get fixed in a population upon the exertion of sustained selection pressure. Selection pressure coupled with genetic diversity drives evolutionary processes leading to herbicide resistance.

A low frequency of plants with reduced sensitivity to glufosinate was observed across a two-stage screening of survivors from a recalcitrant, segregating population. The surviving individuals were described as tolerant because there was no record of their exposure to glufosinate in this field and the plants can be controlled 100% with split application of glufosinate. However, a three-year record, which is what growers can provide generally, is insufficient to assert that the gene regulation demonstrated here is truly ancestral and that it would have been expressed in the total absence of glufosinate selection. *Amaranthus palmeri* is a prolific dioecious plant. Seed could have been transported to the field from elsewhere where glufosinate has been used. Similarly, pollen from other fields with a history of glufosinate use, could have blown through the field and fertilized female plants. Differential gene expression of *CYP72A219* and *GST* and the presence of surviving progeny in intentionally intercrossed populations show that genetic mechanisms exist for the evolution of low-level, or potentially incipient, resistance to glufosinate in some *A*. *palmeri* populations. Vigilance will be required to detect elevated glufosinate tolerance, especially because it is likely multi-genic, impossible to eliminate from populations, and eventually might confer resistance to more than one class of herbicide chemistries and abiotic stresses.
